# Defining diagnostic disclosure in the acute care setting: A concept analysis

**DOI:** 10.1016/j.ijnurstu.2026.105522

**Published:** 2026-04-01

**Authors:** Katherine S. Pitcher, Lauren Massimo, Michael A. Stawnychy, Kathryn H. Bowles

**Affiliations:** a University of Pennsylvania School of Nursing, Philadelphia, PA, USA; b NewCourtland Center for Transitions and Health, Philadelphia, PA, USA; c Leonard Davis Institute for Health Economics, Philadelphia, PA, USA; d University of Pennsylvania, Frontotemporal Degeneration Center, Philadelphia, PA, USA; e Center for Home Care Policy & Research, VNS Health, New York, NY, USA

**Keywords:** Disclosure, Diagnosis, Hospitals, Communication, Patients, Health personnel

## Abstract

**Background:**

Patients are often unaware of their medical diagnoses, impeding their ability to effectively self-manage their conditions. Diagnostic disclosure, the act of clinicians making diagnoses known to patients, can enhance patient awareness, understanding, and adherence to recommended treatments and care plans. Published literature about diagnostic disclosure is limited to dementia, autism, and cancer, and although diagnostic disclosure commonly occurs during hospitalization, what it entails is rarely considered in the acute care setting. Developing a clear definition of diagnostic disclosure in acute care is essential to understand its potential impact. Aim: Develop conceptual and operational definitions of diagnostic disclosure in the acute care setting.

**Methods:**

The Walker and Avant eight-step approach to concept analysis was employed to identify defining attributes, antecedents, and consequences of diagnostic disclosure in the acute care setting. Four databases (PubMed, CINAHL, Scopus, EMBASE) were searched using keywords and controlled vocabulary to identify studies related to diagnostic disclosure among adult populations in the acute care setting. This search yielded 2052 unique articles, of which 37 (published between 1994 and 2025) met inclusion criteria.

**Results:**

No articles that used the term “diagnostic disclosure” provided a definition. Therefore, two defining attributes, *The Disclosure Process* and *Diagnostic Content*, identified in the literature guided the development of conceptual and operational definitions. Diagnostic disclosure is defined as the process through which a clinician names and explains a patient’s diagnosis. Diagnostic disclosure can be measured by the “fullness” of the diagnostic content disclosed. For example, “full” diagnostic disclosure involves both naming and explaining the diagnosis, whereas partial disclosure may either name or euphemistically explain the diagnosis. Model, Borderline, Related, and Contrary Cases further circumscribe the concept. Antecedents included *Clinician Attitudes and Beliefs*, *Clinician Skill*, *Patient Characteristics*, *Patient Curiosity*, *Characteristics of the Diagnosis*, *Cultural Context*, *Family Consent*, *Environment*, and *The Diagnostic Process*. Consequences included *The Patient’s Right, Information-Seeking Patients*, *Shared Decision-Making*, *Grief and Coping*, *Disease Adaptation*, *Strengthened Relationships*, *Adherence and Self-Care*, and *Well-Being and Quality of Life*.

**Conclusions:**

The definitions provided advance our understanding of diagnostic disclosure with implications for practice and future research. A clearly defined concept sets the foundation to measure, predict, and investigate diagnostic disclosure further. This analysis identifies diagnostic disclosure as critical to patient-centered care, with potential to improve communication, enhance self-care, and prevent rehospitalization. Future efforts should focus on testing the operational definition, evaluating effects on patient outcomes, and surveying patients, caregivers, and clinicians to inform clinical integration.

## 1. Background

For many patients, hospitalization is their predominant interaction with the healthcare system (National Academies of Sciences et al., 2018) and is a pivotal opportunity to engage patients in their care (Nassar et al., 2019). During hospitalization, patients may be newly diagnosed with serious illnesses, yet are discharged without a clear understanding of their health status and continuing health needs (Lin et al., 2025). This discordance may compromise their self-care and adherence to treatments and follow-up recommendations, ultimately increasing the risk of rehospitalization (Mabire et al., 2019; Liang et al., 2021; Kosobucka et al., 2022). Clinicians who possess diagnostic knowledge are ethically and professionally obligated to convey health-related information, including diagnoses, to patients. This act of sharing diagnoses with patients is known as diagnostic disclosure.

Diagnostic disclosure exposes overlooked dimensions of foundational nursing theories, such as “awareness” in Meleis’s Transition’s Theory and “reflection” in Riegel’s Theory of Self-Care (Meleis et al., 2000; Riegel et al., 2012). Through disclosure, patients can reflect on experiences that led to hospitalization and begin attributing symptoms to their new diagnosis. Patient awareness of health transitions (i.e., a new diagnosis) is critical for meaningful engagement in that transition (Meleis et al., 2000). Despite the importance of awareness and reflection to self-care and engagement, diagnostic disclosure remains inconsistently practiced and poorly defined, particularly in the acute care (i.e., hospital) setting.

The existing literature and practice guidelines on diagnostic disclosure predominantly focus on conditions such as autism, cancer, and dementia, diagnoses that are often managed in outpatient settings (Gan et al., 2018; Abe et al., 2019; Almog et al., 2023; O’Brien et al., 2024). However, diagnostic disclosure in acute care, where patients often receive new, serious, and/or unexpected diagnoses such as heart failure or sepsis, is seldom discussed (Gallop et al., 2015; McDermott and Roemer, 2021; Shropshire et al., 2023). Similar concepts, such as “disease awareness” (Iroegbu et al., 2022; Finlayson et al., 2024), fail to consider the conditions under which patients *become* aware of their diagnosis.

Physicians and other diagnosticians are typically considered responsible for disclosing diagnoses to patients (Ménard et al., 2025). Nurses report that communicating an initial diagnosis falls outside their scope of practice (Newman, 2016), whereas nurses with prescribing authority (i.e., nurse practitioners), are authorized to disclose diagnoses in countries such as the United States and Canada (College of Nurses of Ontario, 2025). On rare occasions, other clinicians such as laboratory technicians have also disclosed diagnoses to patients (Tinland et al., 2025). Clinicians include prescribing providers (e.g., physicians, nurse practitioners), nurses, and other health professionals involved in patient care (Clinicians, 2024). Not all clinicians bear the duty to disclose diagnoses, yet many still play essential roles in the diagnostic disclosure process. For example, nurses advocate for patients, educate, and provide support after the initial disclosure (Newman, 2016). Examining all clinician perspectives is, therefore, necessary to understand diagnostic disclosure and what it entails.

The absence of clear, evidence-based conceptual and operational definitions of diagnostic disclosure in acute care settings hinders clinicians’ ability to meet patients’ critical needs for information, potentially contributing to adverse outcomes after discharge. Despite multiple countries enacting laws to enforce patients’ rights for information over the last 10 to 25 years (U.S. Department of Health and Human Services, Centers for Medicare, and Medicaid Services, 2024; Glauzy, 2025; Patient’s rights in Finland, 2025), the disclosure of many diagnoses, particularly in acute care, remains unaddressed. This gap raises concerns given the potential consequences of lacking diagnostic disclosure. Defining and analyzing diagnostic disclosure may unlock a new opportunity to improve post-discharge outcomes, such as rehospitalization, for patients transitioning home. Therefore, this concept analysis aims to develop conceptual and operational definitions of diagnostic disclosure in the acute care setting.

## 2. Methods

### 2.1. Approach

The Walker and Avant approach was used to analyze the concept of diagnostic disclosure. This approach was chosen because of its systematic process to clarify and define concepts (Walker and Avant, 2005). Because diagnostic disclosure has yet to be conceptually or operationally defined, the Walker and Avant approach is ideal for achieving our aims through its clear step-by-step framework. It includes eight steps: (1) selecting a concept, (2) determining the aim of the analysis, (3) identifying all uses of the concept, (4) determining defining attributes, (5) identifying model cases, (6) borderline, and contrary cases; (7) identifying antecedents and consequences, and (8) defining empirical referents (Walker and Avant, 2005). The results presented below are organized according to Steps 3 to 8 (Walker and Avant, 2005).

### 2.2. Data search

The search strategy was developed in collaboration with a university librarian and adapted for each database: CINAHL, PubMed, Embase, and Scopus. CINAHL was chosen to capture nursing perspectives on the topic. PubMed, Embase, and Scopus were selected to capture all biomedical perspectives relevant to the acute care setting. No time limits were placed on searches, which were conducted on May 23, 2024, and August 4, 2025. Search terms (such as “bad news”, “diagnosis”, “disclosure”, “telling”, “condition”, and “patient awareness”) and controlled vocabulary terms (such as “Truth Disclosure”, “Hospitals”, “Diagnosis”, and “Interpersonal Communication”) were used whenever possible.

### 2.3. Screening

Inclusion criteria were studies of adult populations (age ≥ 18 years) who were hospitalized and were asked about their awareness of their diagnosis or their experience in learning about their diagnosis. Studies were not limited to acute, chronic, or specific conditions. However, populations concerning genetic, maternal, and prenatal diagnoses, as well as patients in emergency room settings, were excluded to maintain focus on disclosure to adult patients in acute care settings receiving treatment for acute and/or chronic conditions that commonly affect aging adult populations.

**Fig. 1.**
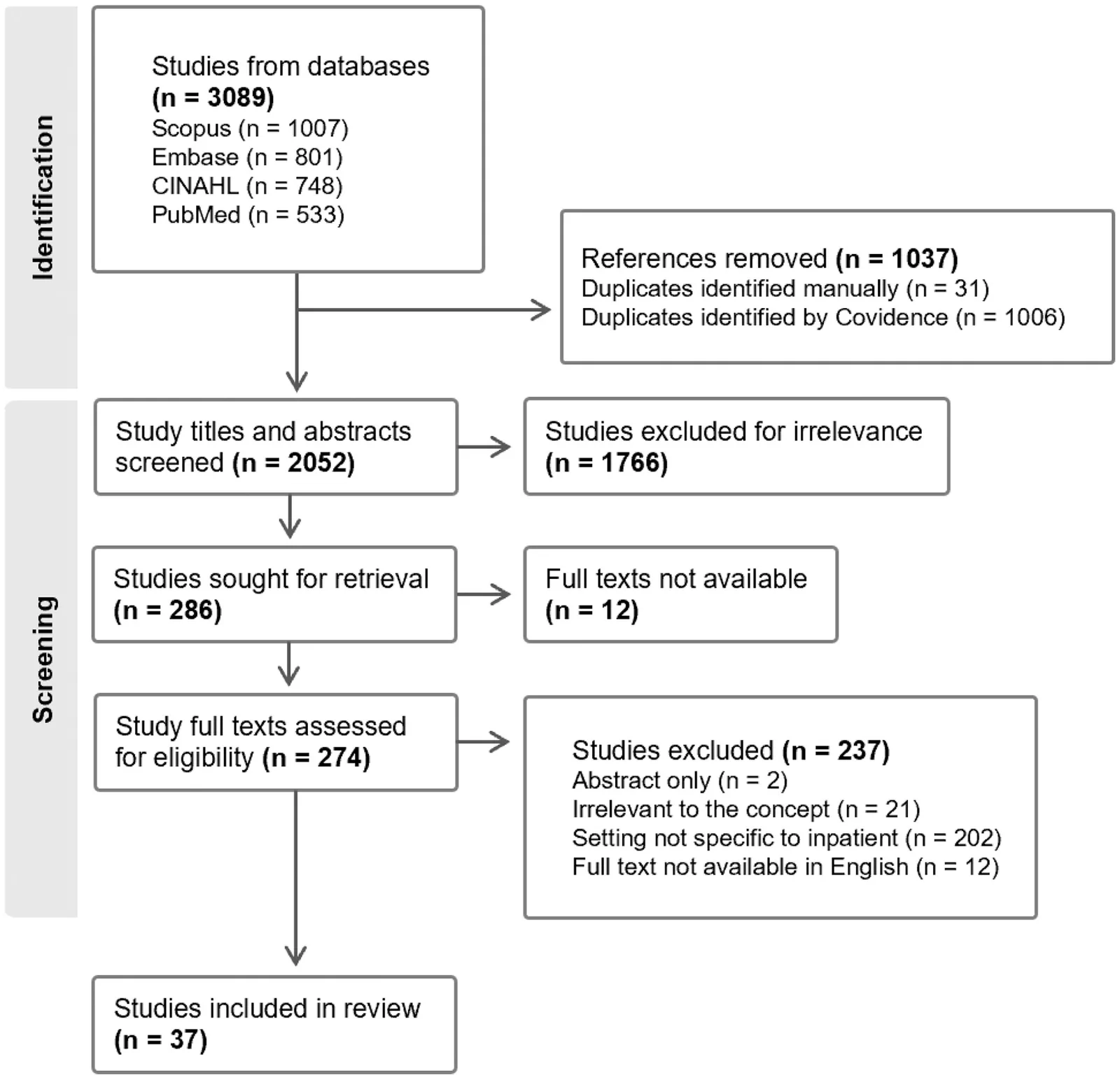
Preferred Reporting Items for the Systematic reviews and Meta-analysis (PRISMA) flow diagram.

One reviewer determined eligibility through title and abstract screening (2052 articles) and full text review (274 articles). Twelve full texts were not available and therefore not retrieved. At this phase, articles were excluded if they only included an abstract (n = 2), were irrelevant to the concept (n = 21), their setting was not specific to the acute care setting (n = 202), or their full text was not available in English (n = 12). See Fig. 1 for a Preferred Reporting Items for Systematic Reviews and Meta-Analyses (PRISMA) flow diagram.

Included texts were read in full using Zotero, a citation management tool that facilitated notetaking and the coding of recurring words, terms, and phenomena. Elements that embodied or explained diagnostic disclosure were highlighted and coded as potential defining attributes. Circumstances that “must occur or be in place” preceding diagnostic disclosure were coded as potential antecedents, and events “that occur as a result of” diagnostic disclosure were coded as potential consequences (Walker and Avant, 2005, p. 178). Initial descriptive codes were organized using Lucid Chart, where related concepts were clustered and redundancies were removed. Attributes, antecedents, and consequences were iteratively refined, collapsed, and re-categorized during this process. Conceptual mapping was also utilized to visualize successive events, distinguishing antecedents, defining attributes, and consequences from each other (All and Huycke, 2007).

Additionally, each study was graded according to Polit and Beck’s evidence hierarchy to categorize study types, such as observational (V), qualitative/descriptive (VII), or non-research sources (VIII) and thereby assess the strength of their evidence (Polit and Beck, 2021) (see Table 1).

## 3. Results

### 3.1. Article characteristics

**Fig. 2.**
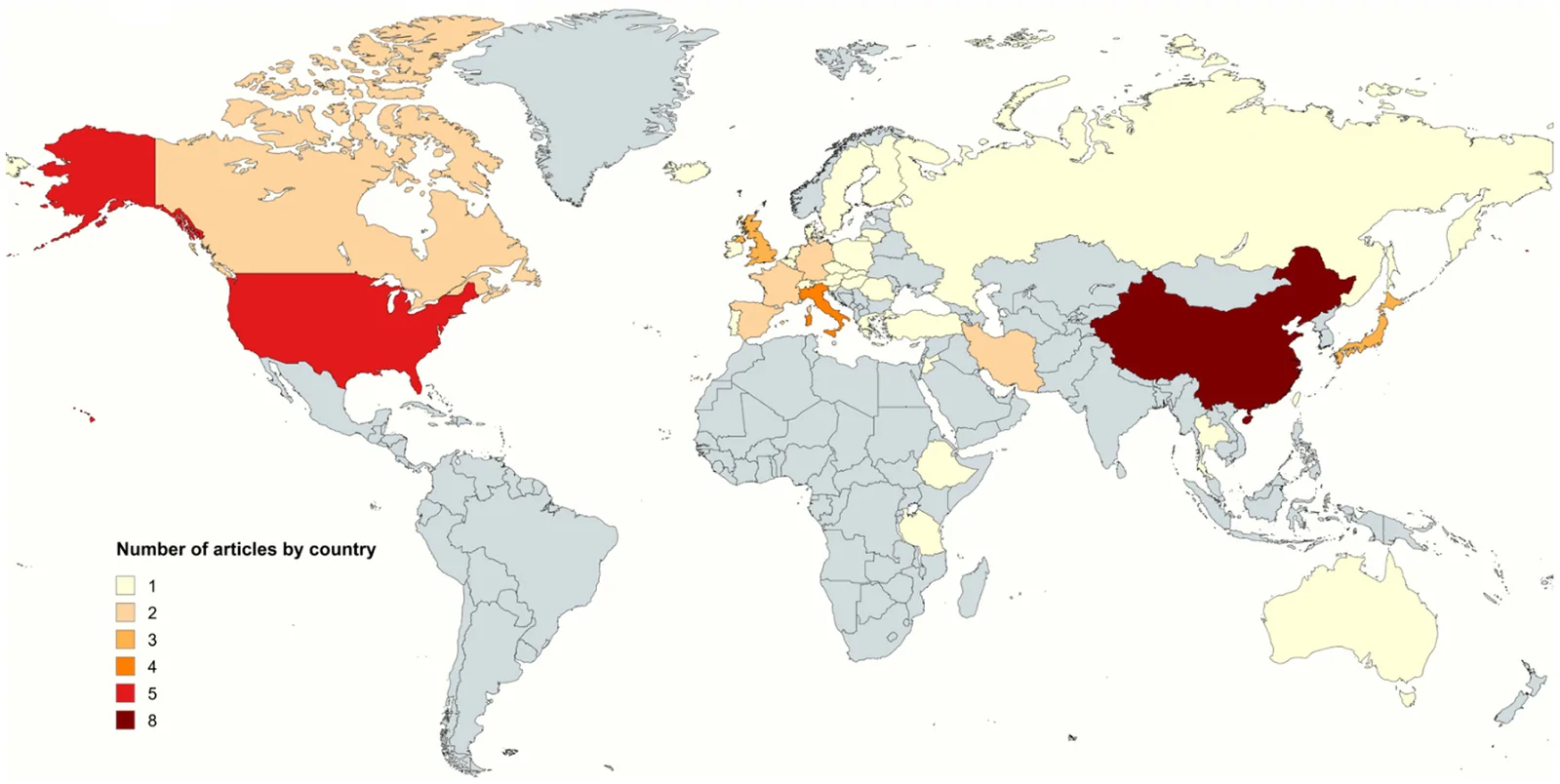
Geographic distribution of included articles.

Search results from four databases retrieved 2052 unique articles after removing 1037 duplicates. Thirty-seven articles met the inclusion criteria; see Table 1 for article characteristics. These articles spanned from 1993 to 2025 and were from 20 different countries; see Fig. 2 for the specific nationalities represented. China was the most common country (8 articles), followed by the United States (5 articles), Italy (3 articles), and Japan (3 articles). One study examined data from 24 different European countries. Included articles examined nine different patient populations, of which cancer was most common (20 articles), followed by chronic kidney disease (3 articles). Eligible articles utilized a variety of study designs; 13 were classified as non-randomized or observational studies (V), 21 as descriptive studies (VII; of which seven were qualitative, two utilized mixed methods, and 13 were quantitative), and two as non-research sources (VIII; a case study and textbook excerpt).

### 3.2. Uses of the concept – Step 3

Only six articles mentioned or discussed the concept by naming it specifically; i.e., by using the term “diagnosis disclosure” or “diagnostic disclosure”. When mentioned, the concept concerned mental health or cancer diagnoses. None of these six articles defined this concept (highlighting the timeliness and necessity of this analysis). The disclosure of other chronic conditions, like chronic kidney disease, coronary artery disease, and heart failure, was not discussed in this fashion and instead framed as patient “awareness” or “illness awareness”. Other studies used language such as disclosing “bad news”, “delivering difficult news”, and “truth-telling”.

### 3.3. Defining attributes – Step 4

Defining attributes are essential characteristics consistently associated with the concept and provide a comprehensive understanding (Walker and Avant, 2005). Two defining attributes, *The Disclosure Process* and *Diagnostic Content*, characterize diagnostic disclosure. *The Disclosure Process* was the most frequent defining attribute among the included articles (Table 2).

#### 3.3.1. Defining attribute 1. The disclosure process

The process of communication and transfer of information between clinicians and their patients is the first defining attribute of diagnostic disclosure. This communication is typically described as being held purposefully, demonstrating respect and sensitivity for the patient. The literature describes how patients are engaged, become aware, develop an understanding of their diagnosis, and/or are adequately informed through this process. Included studies also discuss that diagnostic disclosure epitomizes patient-centered care when the conversation is tailored to the patient receiving the diagnosis. The literature also highlights that the disclosure process should ideally balance efficiency with thoroughness, ensuring it is not rushed yet remains succinct.

Included studies also acknowledge that the disclosure process can be complex and ineffective at times. Frank, abrupt, and even overwhelming and traumatic disclosure conversations are noted. Some promote an encouraging tone and the maintenance of hope to discourage a traumatic disclosure experience for the patient. Patients report a preference for a gradual process of diagnostic disclosure by leading into the conversation or alluding to diagnostic results. Patients may have difficulty comprehending a new diagnosis; two studies point out the importance of *The Process* for patient comprehension of information. This may involve reinforcing disclosure through repeated or iterative conversations, depending on *Patient Characteristics* and *Grief and Coping* (see Antecedents and Consequences – Step 7).

#### 3.3.2. Defining attribute 2. Diagnostic content

The content communicated to patients during the disclosure process is considered as crucial as how it is conveyed, typically focusing on the most important information patients need to know. This content may utilize specific language, detail, explanation, and description to convey the seriousness and stage of the diagnosis truthfully and clearly. Diagnostic disclosure is described as the first step towards patient education, setting the foundation for future learning. However, a balance of disclosing hopeful (positive) and truthful (negative) aspects of the diagnosis is considered important to maintain hope. Including tangible details such as descriptions of anatomical structures and their dysfunction may also enhance patient understanding.

The amount of content disclosed reportedly varies but the concept of an “adequate” or “correct” amount of disease-specific information (such as symptoms, causes, implications, and treatments) came up often in the literature. Comprehensiveness determines the “fullness” of the disclosure; for example, partial diagnostic disclosure involves less or inadequate disclosure (see Step 6). This range of disclosure’s “fullness” informs diagnostic disclosure’s operational definition. Naming the exact diagnosis is particularly important for “full” diagnostic disclosure, as it is considered to be crucial by many articles. Although euphemisms are acceptable in some studies, avoiding euphemisms and alternative terms for the diagnosis is considered to make for a “fuller” diagnostic disclosure. Although avoiding euphemisms is crucial when communicating the name of the diagnosis, one article recommends avoiding medical jargon while explaining the diagnosis and what it means.

These two defining attributes, *The Disclosure Process* and *Diagnostic Content*, set the foundation for defining diagnostic disclosure. Many studies used the word “adequate” to describe diagnostic disclosure, such as adequate information, description, explanation, or disclosure, or to be adequately informed. The prevalence of this descriptor inspires questions about what “adequate” disclosure or information entails. Similarly, the words “full” or “fully” are also used frequently to describe disclosure, patient understanding, being informed, or diagnoses being revealed. While determining the fullness or adequacy of patient understanding or details shared encounters difficulties, fully or adequately revealing or disclosing diagnoses to patients is easier to conceptualize. Therefore, based on the defining attributes presented, diagnostic disclosure is the process through which a clinician shares diagnostic information—specifically, the name of their diagnosis and what it means—with the patient. The amount of diagnostic information communicated determines the “fullness” of diagnostic disclosure; full diagnostic disclosure must include two key pieces of diagnostic information: the name of the diagnosis and some explanation of and/or details about associated symptoms or treatments for the diagnosis.

### 3.4. A model case – Step 5

James is admitted to the hospital with a heart failure exacerbation. His nurse noticed that James lacked an understanding about the reason for his hospitalization and suspected he was unaware of his diagnosis. His nurse raised this concern with James’s physician, advocating for a disclosure conversation. After morning rounds, the physician prepared for and initiated a conversation at James’s bedside. She began by asking if he had heard of heart failure before, and James recalled being told he was at risk after his heart attack. Using this as a foundation, the physician carefully told James that he had “heart failure” and explained the severity of his condition. She remained sensitive to James’s emotions and maintained an encouraging tone. James had many questions after the disclosure conversation, and his nurse reinforced the diagnosis and proceeded with heart failure education up until his discharge. Together, James and his care team planned to manage his new diagnosis collaboratively. James was eager to take the necessary steps to control his symptoms and avoid future hospitalizations by engaging in self-care and attending follow-up appointments.

This model case demonstrates all defining attributes of diagnostic disclosure: *The Disclosure Process* by preparing for and initiating a direct conversation and *Diagnostic Content*, including the name of his diagnosis and essential details. Further, James’s physician demonstrated an organized, patient-centered, sensitive, and respectful approach to the disclosure conversation, as described in The Disclosure Process. This model case also illustrates antecedents and consequences of diagnostic disclosure (to be further described in Step 7), such as *Clinician Attitudes and Beliefs*, *Clinician Skill*, *Patient Characteristics*, and *The Diagnostic Process*, as well as *Information-Seeking Patients*, *Shared Decision-Making*, *Grief and Coping*, *Strengthened Relationships,* and *Adherence and Self-Care*.

### 3.5. Other cases – Step 6

#### 3.5.1. Partial disclosure, a borderline case

Partial disclosure is considered a borderline case because it fails to meet one of diagnostic disclosure’s defining attributes, *Diagnostic Content*. For example, Saunders et al. (2015, 2017) measured patient ability to report “kidney problems” among patients diagnosed with chronic kidney disease. When patients can only self-report vague names of their diagnosis, content disclosed during diagnostic disclosure may have been inadequate and omitted the name of their diagnosis, hence diagnostic content is lacking. Other studies discourage using euphemisms, as they are considered inappropriate and inadequate for diagnostic disclosure. For example, the word “schizophrenia” should be used when disclosing a diagnosis of schizophrenia rather than an alternative term or phrase, such as “mental illness”.

#### 3.5.2. Risk disclosure, a related case

Risk disclosure refers to the disclosure of a patient’s risk of developing a diagnosis. Risk disclosure may at times precede diagnostic disclosure and prepare a patient to accept their at-risk diagnosis. For example, a patient with an ST-elevation myocardial infarction who has been informed of their risk of developing heart failure may be more aware of the diagnosis generally and the possibility of developing heart failure in the future, as seen in the presented Model Case. If combined with other antecedents to diagnostic disclosure (such as *Patient Curiosity*), risk disclosure may lead to earlier or fuller diagnostic disclosure. However, risk disclosure differs from diagnostic disclosure because it does not meet the criteria of *Diagnostic Content*, as the information being communicated does not include a definitive diagnosis.

#### 3.5.3. Therapeutic privilege, a contrary case

Therapeutic privilege is a contrary case to diagnostic disclosure and synonymous with diagnostic non-disclosure. Therapeutic privilege is defined as clinicians withholding information from the patient that is considered to be potentially harmful, either emotionally or physically, such as the news of a severe diagnosis (Sims et al., 2022). It contains no defining attributes of diagnostic disclosure and involves purposeful concealment and avoidance of patient-clinician interaction to discuss a patient’s diagnosis. Therapeutic privilege cites more traditional ethical principles, such as the idea that patients must be protected from the diagnosis and any ensuing emotional distress (see Antecedent 6, Cultural Context). Therapeutic privilege may employ family collusion to uphold these cultural beliefs (see Antecedent 7, Family Consent).

### 3.6. Antecedents and consequences – Step 7

#### 3.6.1. Antecedents – Step 7.1

**Fig. 3.**
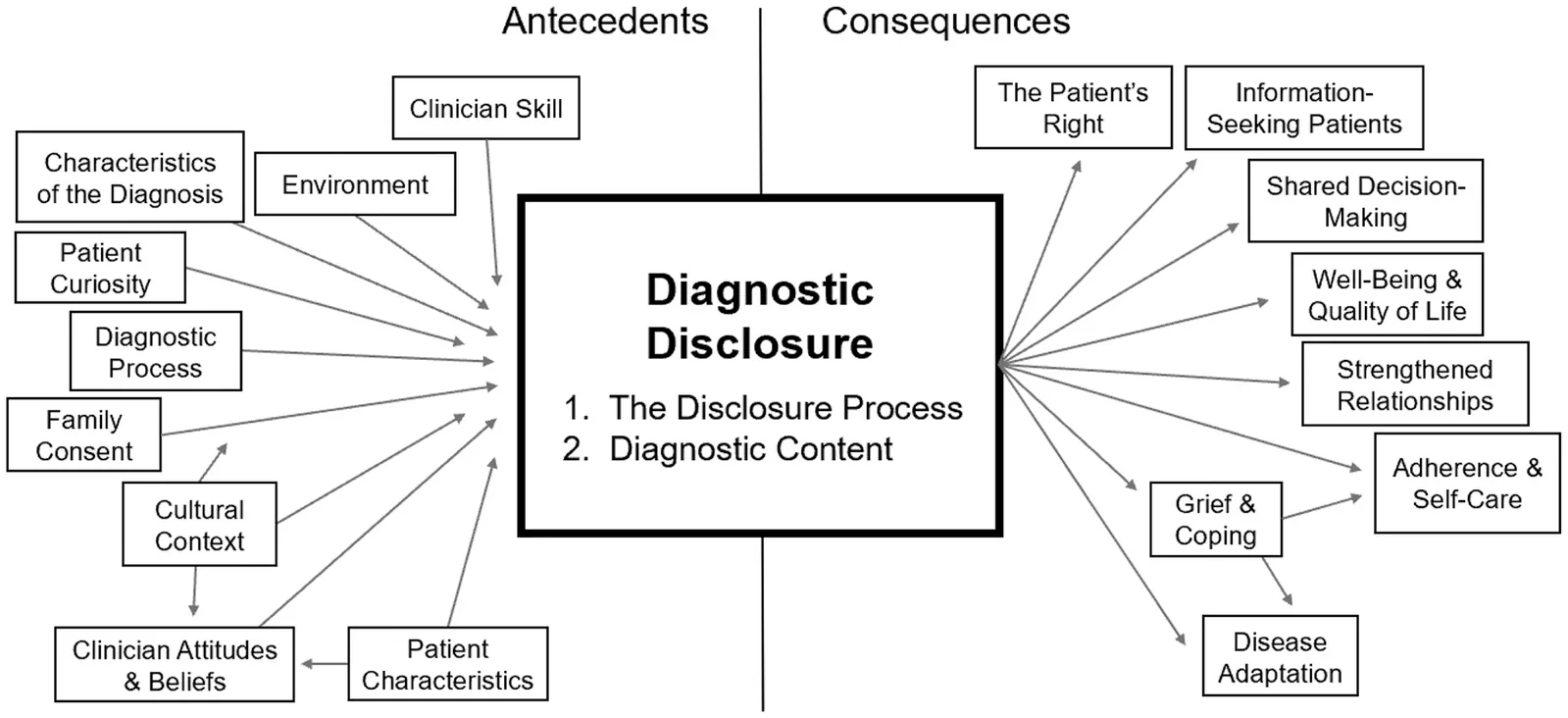
Conceptual model of diagnostic disclosure.

Antecedents are essential conditions or events that must occur or exist before the concept can occur (Walker and Avant, 2005). Nine antecedents were identified in the literature. The most frequent antecedents were *Clinician Attitudes and Beliefs* and *Clinician Skill* (Fig. 3)

##### 3.6.1.1. Antecedent 1. Clinician attitudes and beliefs.

Diagnostic disclosure depends on a clinician’s willingness to disclose the diagnosis, regulated by their empathy, compassion, and honesty. Diagnostic disclosure hinges on the clinician’s perceived importance and subsequent prioritization of this communication, among other necessary topics to be discussed. For example, clinicians who value diagnostic disclosure for patient understanding and quality of life are perhaps more willing to disclose a diagnosis. Some clinicians perceive an obligation to disclose diagnoses to patients, at times due to its relationship with informed consent.

Alternatively, clinician reluctance can inhibit diagnostic disclosure. This reluctance often stems from fear of adverse effects and patient reactions, such as emotional distress or inability to cope. This fear can also lead to incomplete or partial disclosure out of attempts to minimize the ensuing emotional reaction. The literature also suggests that clinicians may fear instilling shame or stigma in patients with stigmatizing diagnoses, such as mental health diagnoses.

##### 3.6.1.2. Antecedent 2. Clinician skill.

In the acute care setting, clinician skill levels in disclosing diagnoses vary widely. A clinician’s communication skills, self-efficacy, and competence in sharing bad news impact the likelihood of diagnostic disclosure. Diagnostic disclosure requires complex communication skills, which can be developed through education and training programs. A clinician’s ability to assess patient understanding, as well as patient preferences, readiness, and educational needs for information, is also a part of *Clinician Skill* and crucially precedes diagnostic disclosure. Without these assessments, clinicians may make incorrect assumptions about the patient’s knowledge, leading to missed opportunities for disclosure.

##### 3.6.1.3. Antecedent 3. Patient characteristics.

Many patient characteristics are found to be associated with diagnostic disclosure, including younger age, married marital status, higher education, and higher socioeconomic status. Prior knowledge also plays a significant role in diagnostic disclosure. When patients are aware of their risk status, they may better understand their potential diagnosis, anticipate that diagnosis during hospitalization, and feel more prepared to accept it. Awareness of comorbidities and pre-conditions can also expedite diagnostic disclosure and make disclosure less shocking, resulting in more informed patients.

A patient’s ability to communicate is a crucial characteristic preceding diagnostic disclosure. A patient’s ability to communicate is complicated by many factors, such as the patient’s clinical stability, cognitive ability, and language proficiency. The clinician’s perception of a patient’s capacity influences their decision to disclose a diagnosis and may be influenced by other patient characteristics, such as age or education. One study found that health literacy was associated with disease-related knowledge; however, when it comes to patient awareness of their diagnosis, another study found no association with health literacy. Included studies suggest that while health literacy supports patient disease-related knowledge, complete unawareness of a diagnosis may be the result of clinicians’ failure to disclose rather than patients’ failure to understand.

##### 3.6.1.4. Antecedent 4. Characteristics of the diagnosis.

The severity of a diagnosis can either facilitate or inhibit diagnostic disclosure. Some studies found that for chronic conditions with insidious onset like chronic kidney disease or heart failure, higher illness severity and poorer organ function may increase the likelihood of disclosure, perhaps due to more frequent healthcare interactions. Conversely, other studies discuss how greater severity may decrease the likelihood or extent of disclosure of sudden, severe diagnoses like late-stage cancer, often due to improbable curability. Some diagnoses are more challenging to diagnose; complex patient presentations can reduce a clinician’s certainty about the diagnosis, and uncertainty in the diagnosis may discourage disclosure, as discussed among psychiatric conditions and chronic kidney disease. Stigmatizing diagnoses, such as mental health diagnoses or even cancer—indigenous translations may equate “cancer” with “worm” or “bug”—are less likely to be disclosed. The visibility of the diagnosis, especially in the case of cancer, can affect the likelihood of diagnostic disclosure, as some cancers are more apparent than others.

##### 3.6.1.5. Antecedent 5. Patient curiosity.

One study discussed how diagnostic disclosure may be more likely if patients are curious or motivated to find out about their diagnosis. Many patients become suspicious of a specific diagnosis and “guess” their diagnosis. If patients are eager for more information, they may pursue a diagnosis by looking for clues, asking questions, or even initiating a conversation with their clinician to confirm their suspicions. However, incongruent informational priorities between the clinician and patient may complicate diagnostic disclosure. For example, patients may prioritize other questions while hospitalized, such as “When am I going home?” rather than asking about their diagnosis, leaving little time for diagnostic disclosure at the bedside. Patient fear of the diagnosis may also lead to avoidance of the disclosure conversation.

##### 3.6.1.6. Antecedent 6. Cultural context.

Culture, shaped by religious, ethical, and regional traditions, informs ethical priorities and thus significantly affects diagnostic disclosure. For example, prior work suggests that Confucian traditions prioritize paternalism and communalism over individual autonomy, discouraging disclosure if the family prefers concealment. Conversely, cultures that prioritize patient autonomy and self-determination tend to promote diagnostic disclosure. Ethical priorities are evolving in traditional societies towards favoring patient autonomy. Nonetheless, the implications of a diagnosis also affect the likelihood of its disclosure; for example, disclosing a grave diagnosis like cancer may even be considered inappropriate in some cultures. It is important to note that different ethical priorities are not generalizable across global hemispheres, continents, or even countries; for example, many parts of Italy embrace more traditional ethical norms over patient autonomy.

##### 3.6.1.7. Antecedent 7. Family consent.

In traditional cultures, family preferences often drive diagnostic disclosure rather than patient rights or preferences. Collaboration between informed family members and clinicians can lead to disclosure, but families often request that clinicians conceal the true diagnosis from the patient. Clinicians may feel they need family consent to disclose the diagnosis, and families may withhold consent due to fears of the patient’s emotional reactions or subsequent psychological distress. This forces clinicians to balance family preferences with their own perceived ethical obligations. When family preferences dominate, diagnostic disclosure is unlikely without patient intervention or *Patient Curiosity*.

##### 3.6.1.8. Antecedent 8. Environment.

Many aspects of the acute care setting are not conducive to diagnostic disclosure. The environment may lack privacy for sensitive conversations, and some clinicians may not have ample opportunities to disclose a diagnosis. Time is crucial to the disclosure process for preparation and conversation; however, time is often limited in the acute care setting due to high patient loads and competing priorities.

##### 3.6.1.9. Antecedent 9. The diagnostic process.

The diagnostic process encompasses several steps that lead to diagnostic disclosure. A diagnosis must be made before diagnostic disclosure, relying on some interactions with clinicians. Symptoms that prompt patients to seek medical help or cause suffering increase the likelihood of diagnostic disclosure. A pre-existing patient-clinician relationship or rapport can also encourage diagnostic disclosure. Hospitalization provides a critical opportunity for diagnosis and disclosure, as increased frequency and volume of patient-clinician interactions increases the chances of disclosure.

#### 3.6.2. Consequences – Step 7.2

Consequences are outcomes or effects following the occurrence of the concept (Walker and Avant, 2005). Seven consequences were identified in the literature. The most prominent consequences were *Shared Decision-Making, Grief and Coping,* and *Adherence and Self-Care*.

##### 3.6.2.1. Consequence 1. The patient’s right.

Diagnostic disclosure is said to fulfill the patient’s legal and moral right to information. Diagnostic disclosure is discussed as a practice that upholds moral principles, such as patient autonomy, enabling patients to make informed decisions about their healthcare. Generally, clinicians view diagnostic disclosure as a moral obligation and the right thing to do. Because of its moral implications, diagnostic disclosure is even mentioned as an indicator of high-quality healthcare. The literature further discusses diagnostic disclosure’s legal implications due to its relationship with informed consent. Legally, informed consent requires that patients be informed of their diagnosis as it pertains to the indication(s) for acute care procedures. Failure to disclose a diagnosis can therefore undermine the validity of informed consent.

The notion that diagnostic disclosure fulfills the patient’s right to be informed transcends cultural contexts and beliefs, as demonstrated in the literature. However, patient rights are not consistently prioritized across all cultures, which can present an ethical dilemma for clinicians of particular cultures, as discussed under relevant antecedents, *Cultural Context* and *Family Consent*.

##### 3.6.2.2. Consequence 2. Information-seeking patients.

Many studies identified how patients seek more detailed information after disclosure and ask questions like, “What should I expect?”. The literature also reports that patients demonstrate eager and positive attitudes after disclosure, welcoming more information and resources about their diagnosis.

##### 3.6.2.3. Consequence 3. Shared decision-making.

Opportunities for shared decision-making after diagnostic disclosure were often discussed in the literature and how they lead to patient engagement, cooperation, more active involvement, and fuller participation in treatment. Although treatment can be initiated without or before diagnostic disclosure, treatment is framed as a collaborative effort after disclosure in the literature, achieved through active discussions between clinicians and patients. A patient informed through diagnostic disclosure is said to be able to fully consent to and participate meaningfully in treatment planning, strategy, and implementation. Patients often demonstrate positive or eager attitudes towards treatment and increased confidence and satisfaction in treatment decisions following diagnostic disclosure. Diagnostic disclosure also increases patients’ locus of control when compared to non-disclosure groups.

##### 3.6.2.4. Consequence 4. Grief and coping.

The literature discussed how patients typically grieve, cope, adjust, and eventually accept their diagnosis. Initial emotional distress, sometimes due to fear of death, is common but usually less severe than clinicians fear. Informed patients tend to experience less cumulative emotional distress than uninformed patients, who may experience more prolonged emotional suffering. Short-term decreases in physical, emotional, social, and role functioning can occur. However, coping, such as through religion, can aid in their adjustment. Diagnostic disclosure also encourages problem-focused coping.

Patients may initially react with shock, disbelief, or denial, which can impede their ability to process the information. A positive diagnostic disclosure experience is vital for patient acceptance of the diagnosis. Emotional distress, decreased functioning, and denial can be mitigated by instilling hope and positivity during the disclosure experience. These points demonstrate the importance of *The Disclosure Process*. Addressing psychosocial needs and providing psychological support for patients struggling during this time is believed to be essential.

##### 3.6.2.5. Consequence 5. Disease adaptation.

The articles included describe a phase of acceptance of and adjustment to the diagnosis following *Grief and Coping*. Diagnostic disclosure facilitates this adjustment, helping patients come to terms with their diagnosis (and any associated stigma, if applicable). This includes making post-discharge changes to their life, such as those related to work, family responsibilities, ongoing treatment, and follow-up arrangements. For some, especially those newly facing terminal diagnoses, this adaptation is described as preparing for death and getting affairs in order. Additionally, some patients may find solace and adaptation through religion.

##### 3.6.2.6. Consequence 6. Strengthened relationships.

Diagnostic disclosure may strengthen relationships between clinicians and patients through trust according to the literature. Communication may become more open, direct, and ongoing after diagnostic disclosure, allowing for more comprehensive discussions of patient concerns and fears, to which clinicians can respond with empathy. Diagnostic disclosure also fosters mutual respect and dignity between clinicians and patients.

Diagnostic disclosure has even been reported to strengthen relationships between family members and patients. Patients report that communication with family members improves after diagnostic disclosure, as they can voice concerns with and seek social support from their family members.

##### 3.6.2.7. Consequence 7. Adherence and self-care.

Disclosure is said to activate and improve both clinical management and self-management of the diagnosis. Including the patient through diagnostic disclosure may enhance patient motivation, self-awareness, and adherence. Diagnostic disclosure may also lead to improved health maintenance activities, such as medication adherence and implementation of lifestyle. However, the effects of diagnostic disclosure on adherence may be moderated by depression. Diagnostic disclosure may also facilitate referrals and follow-up care for patients with chronic kidney disease. Notably, the patient’s ability to attribute their symptoms and experiences to their condition depends on diagnostic disclosure, making diagnostic disclosure necessary to monitor and manage their condition. Further, informed patients tend to attribute the causes of their disease to internal causes, such as distress and lack of awareness, as opposed to uninformed patients, who attribute causes to external forces, such as their environment.

##### 3.6.2.8. Consequence 8. Well-being and quality of life.

Some studies found that diagnostic disclosure is associated with and even leads to improved quality of life. Diagnostic disclosure alleviates uncertainty and fear, providing peace of mind to patients. The results suggest that it may improve symptom and pain control, patient satisfaction, and self-esteem. Additionally, patients who experience diagnostic disclosure report fewer post-traumatic stress symptoms. One study found no negative impact on spiritual health following diagnostic disclosure. Furthermore, family members of fully informed patients experience less stress and psychological distress compared to those of uninformed patients.

### 3.7. Empirical referents – Step 8

An empirical referent is an observable event that indicates the concept’s occurrence in the real world. An empirical referent of diagnostic disclosure frequently referenced in the literature is a patient’s ability to accurately self-report their diagnosis. This empirical referent is easily measurable and recognizable, which helps identify patients who have experienced diagnostic disclosure in practice. This ability is closely related to the defining attributes of diagnostic disclosure because it indicates that a patient has been informed about their diagnosis by a clinician and received clear communication about, at the very least, the name of their condition.

## 4. Discussion

This analysis successfully developed a conceptual definition of diagnostic disclosure in the acute care setting, advancing our understanding of the concept by identifying its defining attributes, antecedents, and consequences through a rigorous and comprehensive literature search and concept analysis. This concept analysis defines diagnostic disclosure in the acute care setting as a process through which a clinician names and explains a patient’s diagnosis, fulfilling the patient’s right to be adequately informed. Antecedents and consequences discussed in this analysis contextualize diagnostic disclosure by describing necessary existing conditions and significant outcomes associated with the concept. Further, borderline cases and defining attributes, particularly *Diagnostic Content*, inform the operational definition of diagnostic disclosure. *Diagnostic Content* disclosed by the clinician may vary in comprehensiveness, which affects the fullness of disclosure. *Diagnostic Content* describes how both naming *and* explaining the diagnosis are crucial for informing patients of their diagnosis. The conceptual definition describes “full” diagnostic disclosure. Other situations of diagnostic disclosure may be partial—in which the explanation of the patient’s condition is inadequate for their understanding—or euphemistic—in which the name of the diagnosis is omitted, but the diagnosis is described. For example, “sepsis” may not be named, but the patient could understand they had a “very serious infection” or “blood infection.” The operational definition is ready for preliminary use and measurement of the concept in the acute care setting and complements the conceptual definition.

This analysis supports and advances existing literature on diagnostic disclosure. While previous definitions, such as “the act of informing patients of the name and nature of their illness” (Mei and Tu, 2021, p. 1) align with this definition, they lack context, evidence, and acknowledgment of the moral and ethical antecedents and consequences of diagnostic disclosure. Though previous research (Finlayson et al., 2024) has explored similar concepts, such as “awareness of disease status,” investigating diagnostic disclosure allows for a richer understanding by critically examining the procedure of sharing a diagnosis. A significant step towards improving diagnostic disclosure involves adopting consistent terminology in the literature, beginning with this term “diagnostic disclosure.”

### 4.1. Implications for practice and policy

The definition of diagnostic disclosure developed through this analysis has several implications for practice. Acute care clinicians can apply this definition by reflecting on their attitudes and beliefs surrounding diagnostic disclosure and assessing patients’ understanding of their diagnosis to avoid harmful assumptions. Clinicians can also work towards fostering a healthcare environment in which patients are better informed, more engaged, and more confident in their decision-making. The development and use of a “dot phrase” (a tool available in many electronic health records to quickly enter text) would indicate when a disclosure conversation occurs with a patient to prevent diffusion of responsibility in a clinical setting where clinicians rotate patient populations. We suggest that clinical settings should include diagnostic disclosure on the bedside nurse rounding checklist to document the status of diagnostic disclosure and prompt discussion of the appropriate timing and approach during interdisciplinary rounds.

By involving discussions of diagnostic disclosure in multidisciplinary team rounds, diagnostic disclosure becomes a collaborative effort across the whole care team. The Model Case presents an exemplary situation, though real-world scenarios are understandably less seamless. In practice, the decision to disclose can be ethically and clinically complex. Including diagnostic disclosure as a topic during multidisciplinary rounds provides a structured opportunity for addressing disagreements about the decision or timing of disclosure, and for consulting ethics teams as needed. Nurses, in particular, play a critical role in this process. As patient advocates with strong assessment skills and close proximity to patients, nurses are well-positioned to evaluate patient awareness of their diagnosis and identify gaps in understanding (see Clinician Skill). When such gaps are present, nurses can advocate for diagnostic disclosure, provide support, and reinforce key aspects of post-discharge care, including answering patient questions, supporting emotional adjustment, and emphasizing the importance of follow-up care or self-management, as indicated. Ultimately, diagnostic disclosure is a shared clinical responsibility shaped by ongoing communication and patient-centered advocacy.

This definition provides a foundation for developing best practice guidelines and clinical decision support systems for clinicians to disclose diagnoses in the acute care setting. Integrating this definition into educational programs through skill-building and communication training for new and experienced clinicians and clinicians-in-training can empower them to disclose diagnostic information more readily and effectively. Promoting diagnostic disclosure offers an exciting opportunity to advance patient-centeredness and improve health outcomes, as well as to inform the next generations of clinicians.

Diagnostic disclosure becomes highly relevant in light of current policy initiatives in the United States. The 21st Century Cures Act was signed into law in 2016 and is currently being implemented (U.S. Department of Health and Human Services, Centers for Medicare, and Medicaid Services, 2024). As part of this legislation, the information blocking rule (commonly known as “open notes”) took effect in 2021 and mandates that patients have immediate access to clinician clinical notes, facilitating patient engagement in their care (Salmi et al., 2021). While open notes and diagnostic disclosure share overarching end-goals—adherence to recommendations, shared decision-making, engagement in follow-up—open notes raise concerns about patients accessing diagnostic information before clinicians can lead disclosure conversations (Salmi et al., 2021). This highlights the importance and timeliness of investigating diagnostic disclosure; open notes may encourage clinicians to disclose diagnoses in a more timely and structured manner.

This concept analysis was specific to the acute care setting; however, this definition may be generalizable to other healthcare settings, such as primary care and emergency room settings, based on the broad nature of this developed definition. In particular, *The Environment* may be slightly different in outpatient or emergency room settings. Testing this definition in other healthcare settings is encouraged prior to utilization, but a different definition may not be necessary for application in other settings.

### 4.2. Implications for future research

One particularly noteworthy consequence of diagnostic disclosure identified in this analysis is self-care. Patient behaviors described under *Adherence and Self-Care* can be organized according to dimensions of self-care discussed in The Middle-Range Theory of Self-Care (Riegel et al., 2012). For example, medication adherence and lifestyle modifications are both types of self-care maintenance (Riegel et al., 2012). This theory emphasizes the importance of patient reflection and knowledge acquisition for effective self-care (Riegel et al., 2012). Diagnostic disclosure presents a critical opportunity for enhancing patient knowledge and reflection, leading to improved self-care and potentially reducing hospital readmissions (Toukhsati et al., 2019). Riegel’s theories provide a solid theoretical foundation for future research into how diagnostic disclosure affects self-care and patient outcomes.

This concept analysis revealed significant gaps in the literature concerning the strength of evidence and the variety of patient populations studied. The existing literature lacks higher-level evidence on diagnostic disclosure, such as cohort studies or randomized controlled trials. Our analysis also found that only six studies explicitly mentioned “diagnosis disclosure” or “diagnostic disclosure.” These studies focused on mental health and cancer diagnoses, while the disclosure of other chronic conditions, such as chronic kidney disease, coronary artery disease, and heart failure, was largely undiscussed and instead described as “patient awareness” of their diagnosis. These patient populations often face challenges in self-care, self-management, and adherence (Khatib et al., 2019; Du et al., 2022; Niriayo et al., 2024), which can lead to poorer patient outcomes (Toukhsati et al., 2019; Uitvlugt et al., 2021). These gaps reinforce the need for future research to investigate the impact of diagnostic disclosure on self-care and patient outcomes, particularly among patient populations vulnerable to rehospitalization. Future research should aim to generate higher-quality evidence to better understand how diagnostic disclosure affects patient outcomes across different clinical contexts.

There are five key areas in which more research is needed on diagnostic disclosure: on patient comprehension, clinician characteristics, mental health sequelae, disease progression, and improving transitions. Patient comprehension after diagnostic disclosure is only briefly addressed in the literature, despite it being fundamental to similar concepts such as informed consent (Richardson, 2013). One study investigated the association of clinician characteristics with diagnostic disclosure and found no differences based on clinician age and length of work experience (Vogliotti et al., 2022). The effect of a clinician’s specific occupation (physician, advanced practice provider, nurse, etc.) on their beliefs, attitudes, and skill is another opportunity for further research.

Findings on diagnostic disclosure’s effect on anxiety and depression are inconsistent. Some studies report minimal effects on the development of anxiety and depression after delivering bad diagnostic news (Atesci et al., 2004; Ardestani et al., 2015). One source suggests that diagnostic disclosure can lead to depression and feelings of anomie (Dégi, 2009). However, other results indicate that patients who undergo diagnostic disclosure experience less stress and a lower likelihood of anxiety and depression (Dégi, 2009; Yang et al., 2018). Another study found no difference in anxiety levels between informed patients and uninformed patients (Yang et al., 2019). Therefore, more work is needed to determine and implement optimal characteristics of *The Disclosure Process*, such as appropriate timing and an optimal approach to the conversation, to mitigate feelings of hopelessness and deleterious effects on mental health.

Diagnostic disclosure may also play a role in slowing disease progression among patients with chronic illness, such as chronic kidney disease, due to its impact on self-management and close relationship with patient education (Saunders et al., 2015). By increasing patient awareness, diagnostic disclosure may lead to fewer complications associated with the diagnosis (Saunders et al., 2015, 2017). Diagnostic disclosure’s close relationship with patient-centered care has led to discussions of its contribution “to a safe transition on hospital discharge” (Olson and Windish, 2010, p. 1302). The connection between timely post-acute care and readmissions among at-risk patient populations such as sepsis survivors (Deb et al., 2019; Oh et al., 2022; O’Connor et al., 2022) and the potential relationship between diagnostic disclosure, treatment, and follow-up care supports this claim.

### 4.3. Limitations

Limitations of this concept analysis must be considered when interpreting and applying the provided definition. Much of the existing literature relies on observational, qualitative, and quantitative descriptive designs, which limits the strength of the evidence provided. No studies were excluded from the analysis based on their literature type. Although the definition was developed considering diverse perspectives, cultures, and diagnoses, our inclusion criteria were limited to full-text articles that were available in English, which may affect the breadth of the review. Additionally, the literature focused almost exclusively on diagnostic disclosure to patients, which is reflected in the proposed definition. It does not address how diagnoses are communicated to next of kin or other family members. Further research is needed to corroborate what diagnostic disclosure entails when other individuals are involved. Despite these limitations, the analysis provides a solid foundation for improving practice and guiding future research in diagnostic disclosure. Addressing these limitations in future studies could lead to a more comprehensive understanding and further refinement of the concept.

## 5. Conclusion

This analysis defines diagnostic disclosure in the acute care setting as the process through which a clinician names and explains a patient’s diagnosis, fulfilling the patient’s right to be adequately informed. Diagnostic disclosure is affected by preexisting attitudes, beliefs, and skill among clinicians; patient characteristics and curiosity; characteristics of the diagnosis; family consent; cultural context; the environment; and the diagnostic process. Diagnostic disclosure may lead to information-seeking patients, shared decision-making, grief and coping, strengthened relationships, adherence and self-care, well-being and quality of life, and disease adaptation. Diagnostic disclosure can be measured as full if the diagnosis is both named and explained and partial if missing either of these components.

## Supplementary Material

Supplementary Table

## Figures and Tables

**Table 1:** Table of evidence.

Authors	Country	Level of evidence	Diagnosis	Population	Defining attributes	Antecedents	Consequences
Andruccioli et al., 2007	Italy	VII	General (hospice setting)	Patients	Disclosure Process, Diagnostic Content	Clinician Beliefs; Cultural Context; Characteristics of the Diagnosis	Shared Decision-making; Strengthened Relationships; Well-Being and Quality of Life; Disease Adaptation
Ardestani et al., 2015	Iran	VII	Cancer	Patients	Disclosure Process, Diagnostic Content	Clinician Attitudes and Beliefs; Clinician Skill	Information-seeking Patients; Shared Decision-making; Well-Being and Quality of Life; Disease Adaptation
Atesci et al., 2004	Turkey	VII	Cancer	Patients	Disclosure Process	Cultural Context	Grief and Coping; Mental Health Sequelae; Self-care and Adherence; Disease Adaptation
Athanas, Gasto, & Renatha, 2020	Tanzania	VII	General (terminal illness)	Patients, clinicians	Disclosure Process, Diagnostic Content, The Patient’s Right	Clinician Skill; Cultural Context; Family Consent; Environment	Information-seeking Patients; Strengthened Relationships; Disease Adaptation
Cavanna et al., 2009	Italy	VII	Cancer	Patients	Disclosure Process, Diagnostic Content	Cultural Context	Information-seeking Patients; Shared Decision-making; Grief and Coping
Centeno-Cortés & Núñez-Olarte, 1994	Spain	VII	Cancer (terminally ill)	Patients	Disclosure Process, Diagnostic Content	Clinician Attitudes and Beliefs; Clinician Skill	Information-seeking Patients; Shared Decision-making; Grief and Coping; Strengthened Relationships
Cho, 2018	USA	VIII	Borderline personality disorder	Physicians	Disclosure Process, Diagnostic Content	Clinician Attitudes and Beliefs	Shared Decision-making; Strengthened Relationships; Adherence and Self-care
Cleary, Hunt, & Walter, 2010	Australia	VII	Mental health diagnoses	Clinicians	Disclosure Process, Diagnostic Content	Clinician Attitudes and Beliefs; Clinician Skill; Characteristics of the Diagnosis; Environment	The Patient’s Right, Information-seeking Patients; Shared Decision-making; Strengthened Relationships; Adherence and Self-care; Well-Being and Quality of Life
Dégi, 2009	Romania	VII	Cancer	Patients	Disclosure Process	Clinician Skills; Patient Characteristics; Characteristics of the Diagnosis; Cultural Context; Family Consent	Shared Decision-making; Grief and Coping; Adherence and Self-care; Well-Being and Quality of Life; Disease Adaptation
Gallop et al., 2015	UK (London) & US (Alabama)	VII	Severe sepsis	Patients, caregivers	Disclosure Process, Diagnostic Content	Patient Characteristics	The Patient’s Right; Information-seeking Patients; Grief and Coping; Adherence and Self-care
Gessesse et al., 2024	Ethiopia	VII	General	Patients and physicians	The Disclosure Process, Diagnostic Content	Clinician Skill; Characteristics of the Diagnosis; Cultural Context; The Environment	The Patient’s Right; Information-Seeking Patients; Shared Decision-Making; Grief and Coping; Strengthened Relationships; Adherence and Self-Care;
Hosaka et al., 1999	Japan	VII	Cancer	Patients	Disclosure Process, Diagnostic Content	Clinician Attitude and Beliefs; Family Consent	The Patient’s Right; Strengthened Relationships
Huang et al., 2016	China	VII	Cancer	Patients	Disclosure Process	Clinician Skill; Patient Curiosity; Characteristics of the Diagnosis; Cultural Context	Information-seeking Patients; Shared Decision-making; Grief and Coping
Jie et al., 2015	China	V	Cancer (liver)	Patients	Disclosure Process	Patient Characteristics; Cultural Context; Family Consent	The Patient’s Right; Shared Decision-Making; Well-Being and Quality of Life; Adherence and Self-care
Jie et al., 2020	China	V	Cancer (liver)	Patients	Disclosure Process, Diagnostic Content	Clinician Attitudes & Beliefs; Patient Curiosity; Patient Characteristics; Family Consent	Shared Decision-making; Grief and Coping; Strengthened Relationships; Well-Being and Quality of Life; Disease Adaptation
Kaufert, 1999	Canada	VIII	Cancer	Patients (Aboriginal)	Disclosure Process, Diagnostic Content	Clinician Attitudes and Beliefs; Patient Characteristics; Characteristics of the Diagnosis; Cultural Context; Family Consent; The Diagnostic Process	Shared Decision-making
Kawakami et al., 2001	Japan	VII	Cancer	Patients (older adults)	Disclosure Process, Diagnostic Content	Clinician Attitudes and Beliefs; Patient Characteristics; Characteristics of the Diagnosis; Cultural Context; Family Consent; Environment	Shared Decision-making
Lainščak et al., 2007	24 different European countries	V	Heart Failure	Patients	Diagnostic Content	Characteristics of the Diagnosis	Adherence and Self-care
Lheureux et al., 2022	France	V	Cancer (lung)	Patients	none	none	Grief and Coping
Li et al., 2012	China	VII	Cancer	Patients, family members	Disclosure Process	Cultural Context; Family Consent	The Patient’s Right; Shared Decision-making
Lin, 1999	Taiwan	V	Cancer	Patients	Disclosure Process	Clinician Skills; Patient Characteristics; Family Consent; Diagnostic Process	The Patient’s Right; Shared Decision-making; Well-Being and Quality of Life; Strengthened Relationships
May, 1993	Scotland	VII	General (medical-surgical setting)	Nurses	Disclosure Process, Diagnostic Content	Clinician Attitudes and Beliefs; Clinician Skill; Patient Characteristics; Family Consent; The Diagnostic Process	Shared Decision-Making; Grief and Coping; Strengthened Relationships; Adherence and Self-care
Miles et al., 2025	Jordan	VII	General	Physicians	The Disclosure Process, Diagnostic Content	Clinician Attitudes and Beliefs; Clinician Skill; Cultural Context; Family Consent; The Environment	Shared Decision-making
Nadeau et al., 2021	Vancouver, Canada	VII	Traumatic spinal cord injury (tSCI)	Patients, family members	Disclosure Process, Diagnostic Content	Clinician Attitudes and Beliefs; Clinician Skill; The Diagnostic Process	Information-seeking Patients; Shared Decision-making; Grief and Coping
Nakanishi et al., 2002	Japan	V	Cancer	Family members	none	Clinician Attitudes and Beliefs; Clinician Skill; Characteristics of the Diagnosis; Cultural Context	Shared Decision-making; Well-Being and Quality of Life
Olson and Windish, 2010	Connecticut, USA	VII	General	Patients and clinicians	Disclosure Process, Diagnostic Content	Clinician Skill	Shared Decision-making; Strengthened Relationships; Adherence and self-care; Well-Being and Quality of Life
Pinyopornpanish et al., 2017	Thailand	VII	Cancer	Patients	none	Family Consent	Shared Decision-making; Grief and Coping; Strengthened Relationships; Adherence and Self-care; Well-Being and Quality of Life
Sadat-Aghahosseini et al., 2012	Iran	VII	Cancer	Patients	none	Clinician Attitudes and Beliefs; Patient Characteristics	The Patient’s Right; Grief and Coping; Well-Being and Quality of Life; Disease Adaptation
Saunders et al., 2015	Chicago, IL, USA	V	Chronic kidney disease (CKD)	Patients	Diagnostic Content	Characteristics of the Diagnosis; Patient Characteristics	Adherence and Self-care
Saunders et al., 2017	Chicago, IL, USA	V	CKD	Patients	Disclosure Process, Diagnostic Content	Patient Characteristics; Patient Curiosity; Characteristics of the Diagnosis; Environment; The Diagnostic Process	The Patient’s Right; Adherence and Self-care; Grief and Coping
Svavarsdeóttir et al., 2023	Iceland	V	Coronary artery disease (CAD)	Patients	Disclosure Process, Diagnostic Content	Clinician Attitudes and Beliefs; Clinician Skill; Patient Curiosity	Grief and Coping; Strengthened Relationships; Adherence and Self-care
Vogliotti et al., 2022	Italy	VII	General (terminal illness)	Physicians	Disclosure Process	Clinician Attitudes and Beliefs; Clinician Skill; Patient Characteristics; The Diagnostic Process	The Patient’s Right; Grief and Coping; Well-Being and Quality of Life
Wagner et al., 2017	Germany	V	CAD with comorbid CKD	Patients	Disclosure Process, Diagnostic Content	Patient Characteristics; Characteristics of the Diagnosis; The Diagnostic Process	Shared Decision-making; Adherence and Self-Care
Yang et al., 2018	China	VII	Cancer (GI)	Patients	Disclosure Process	Clinician Attitudes and Beliefs; Patient Curiosity; Family Consent	Information-seeking Patients; Shared Decision-making; Grief and Coping; Well-Being and Quality of Life; Disease Adaptation
Yang et al., 2019	China	V	Cancer (GI)	Patients	Disclosure Process	Patient Characteristics; Cultural Context	Grief and Coping; Well-Being and Quality of Life
Zhang et al., 2017	China	V	Cancer (esophageal)	Family members	Disclosure Process	Patient Characteristics; Characteristics of the Diagnosis; Family Consent; Cultural Context	Adherence and Self-care; Well-Being and Quality of Life
Zhou et al., 2023	China	V	Mental health diagnoses	Patients, family members	Disclosure Process	Clinician Attitudes; Characteristics of the Diagnosis	The Patient’s Right; Shared Decision-making; Adherence and Self-care

**Table 2:** Details on Defining Attributes, Antecedents, and Consequences.

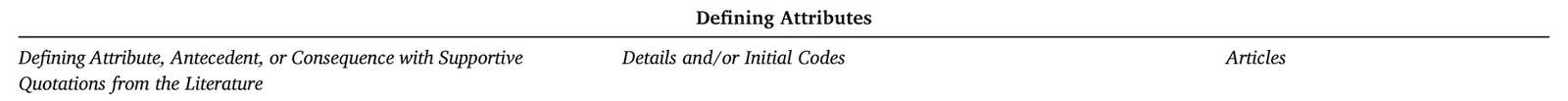
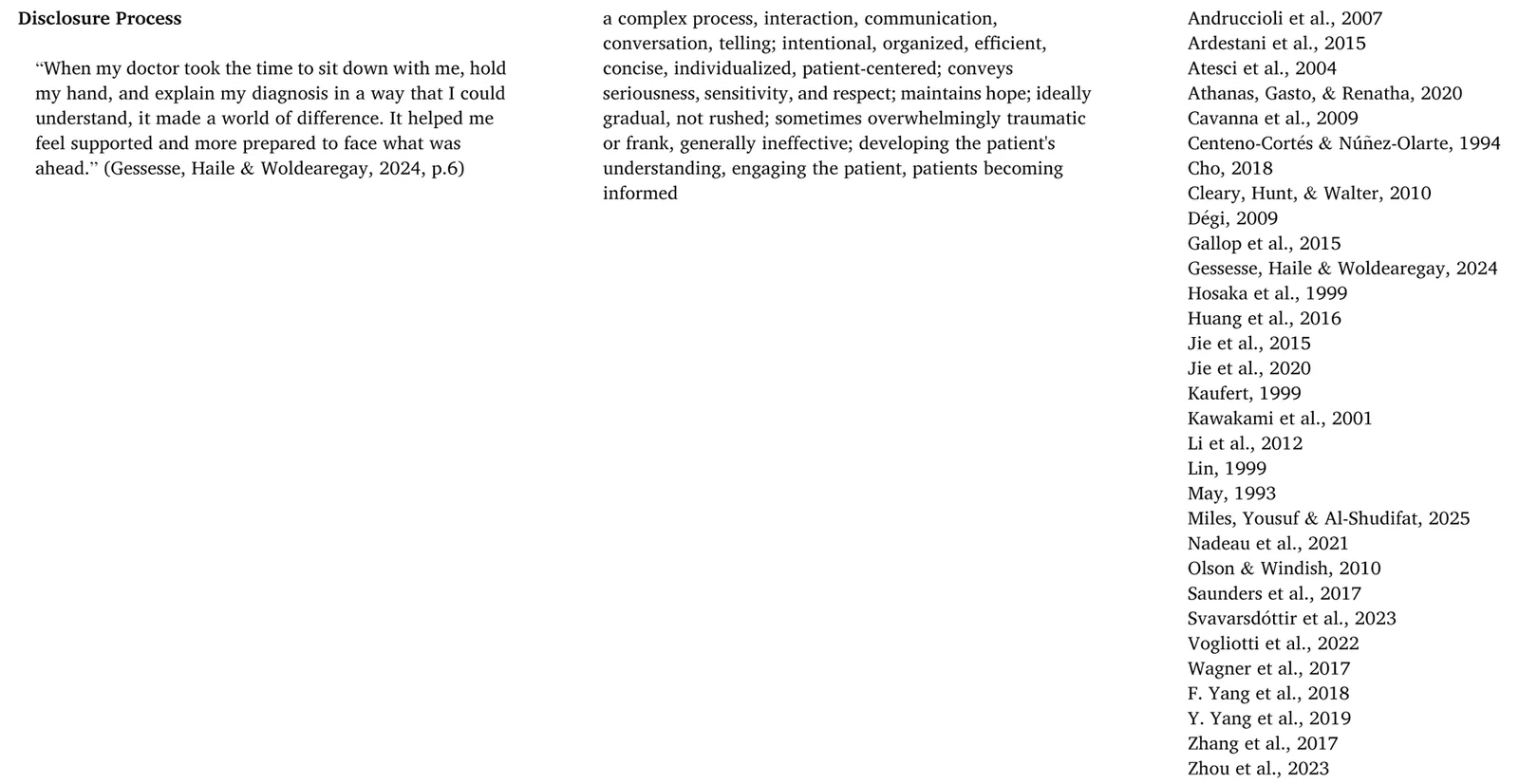
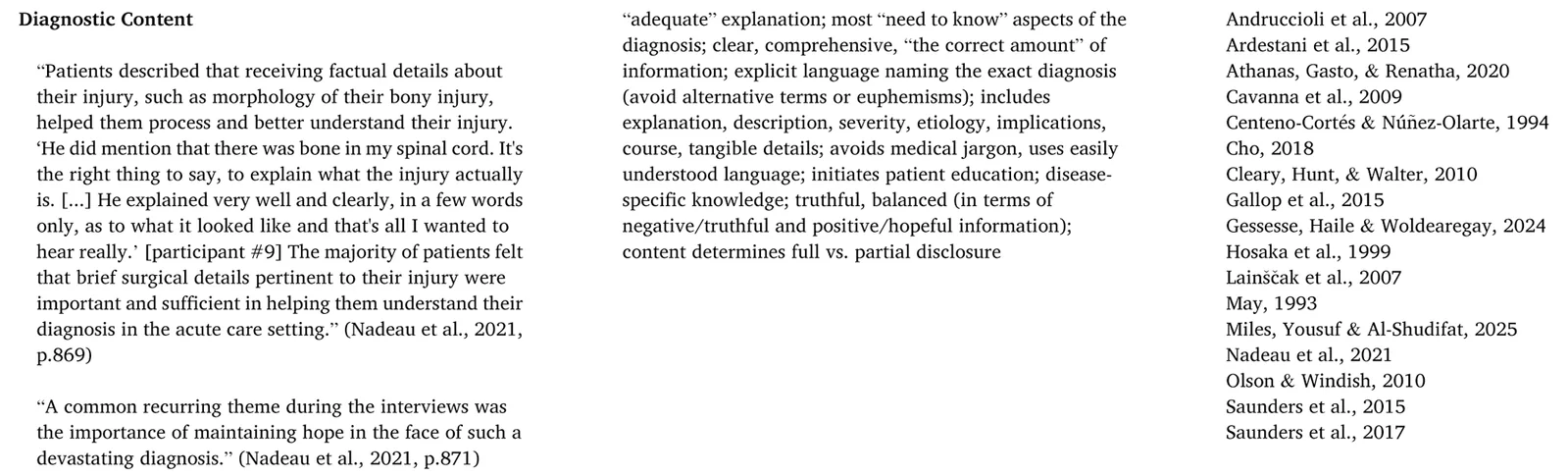
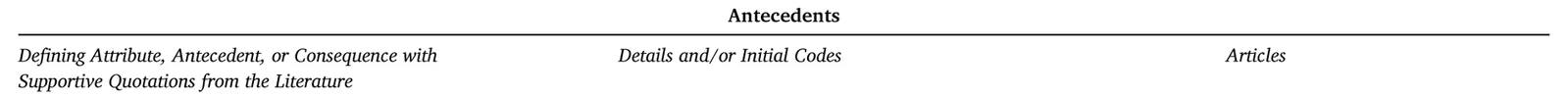
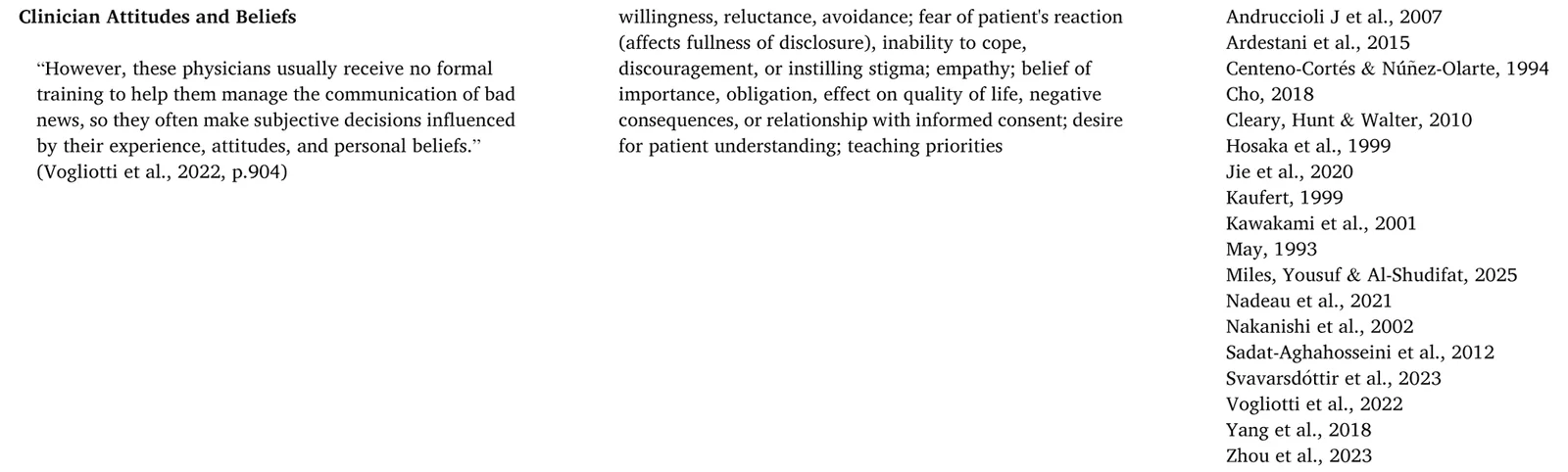
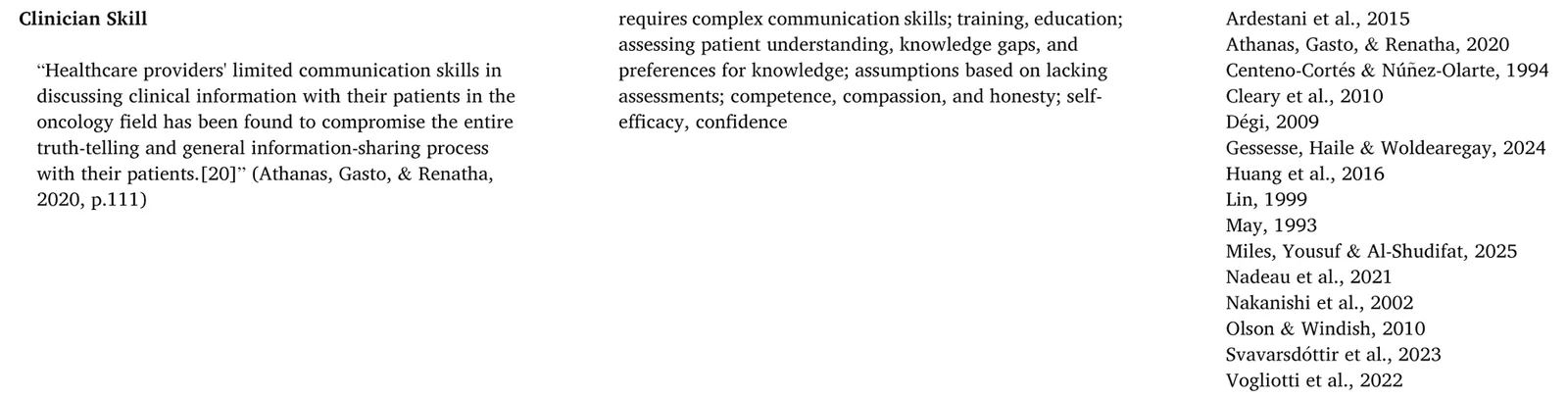
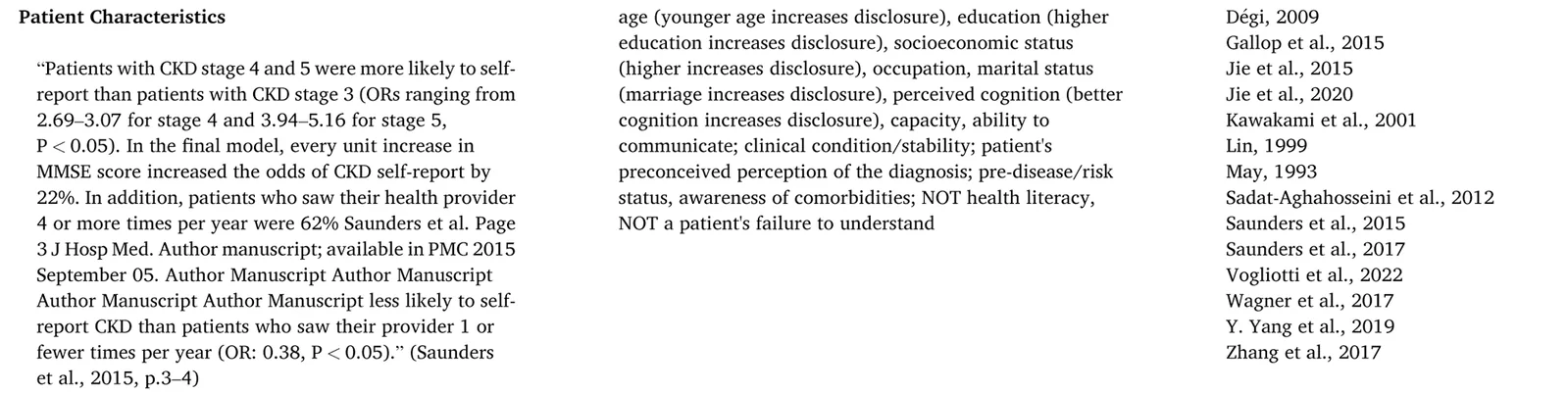
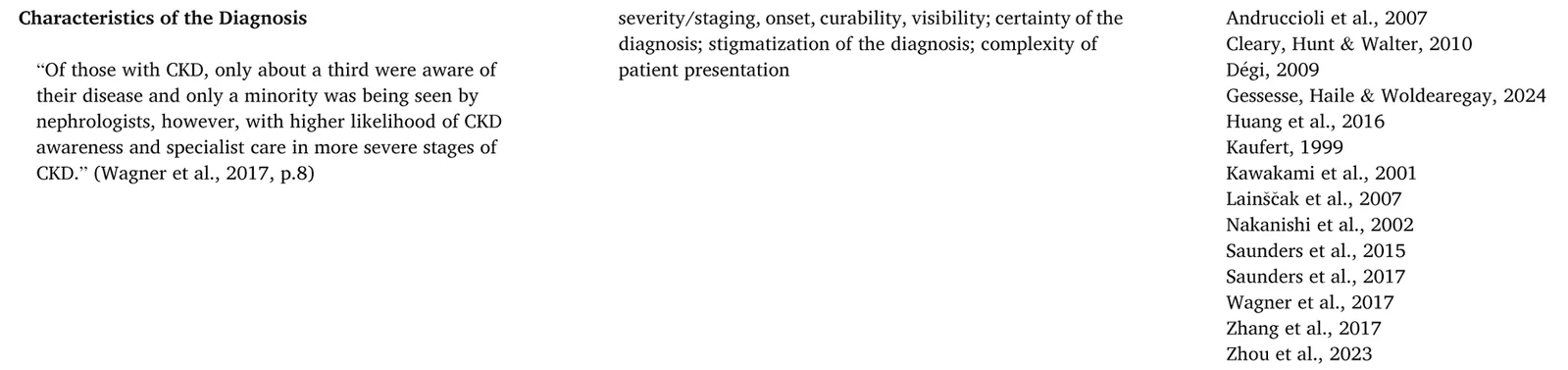
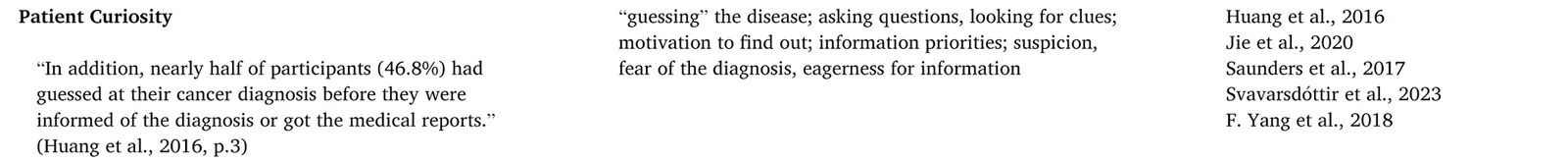
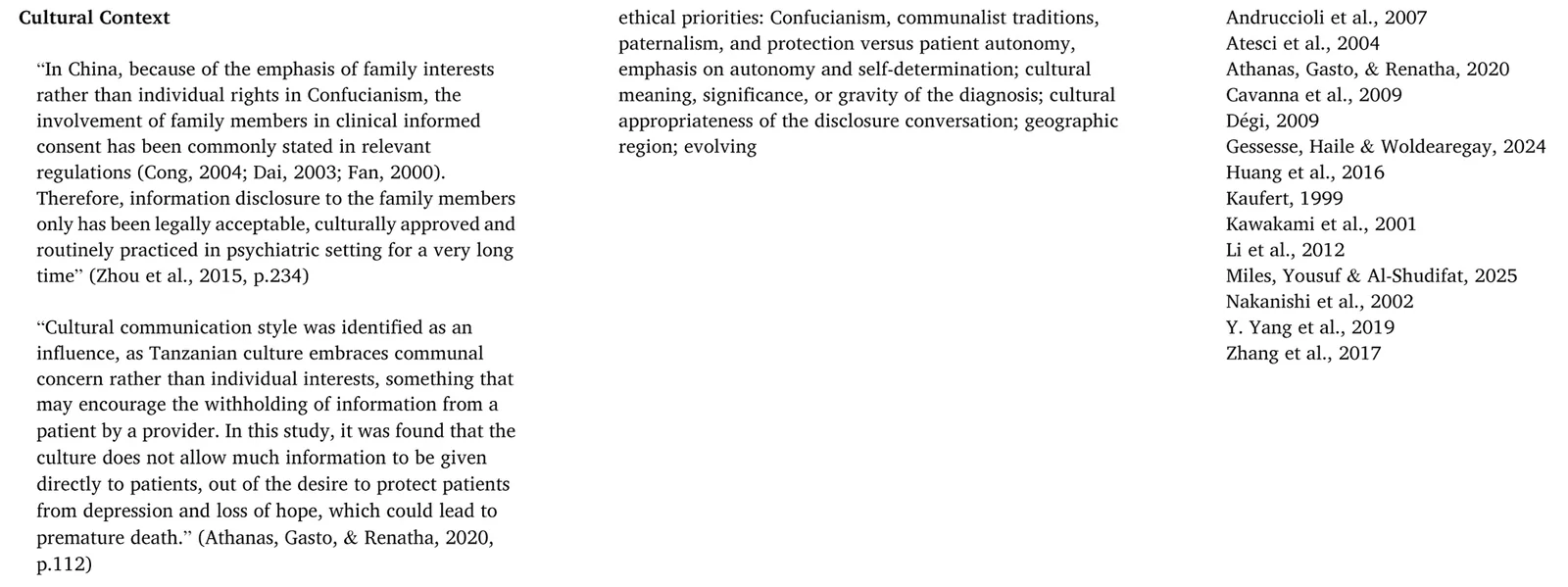
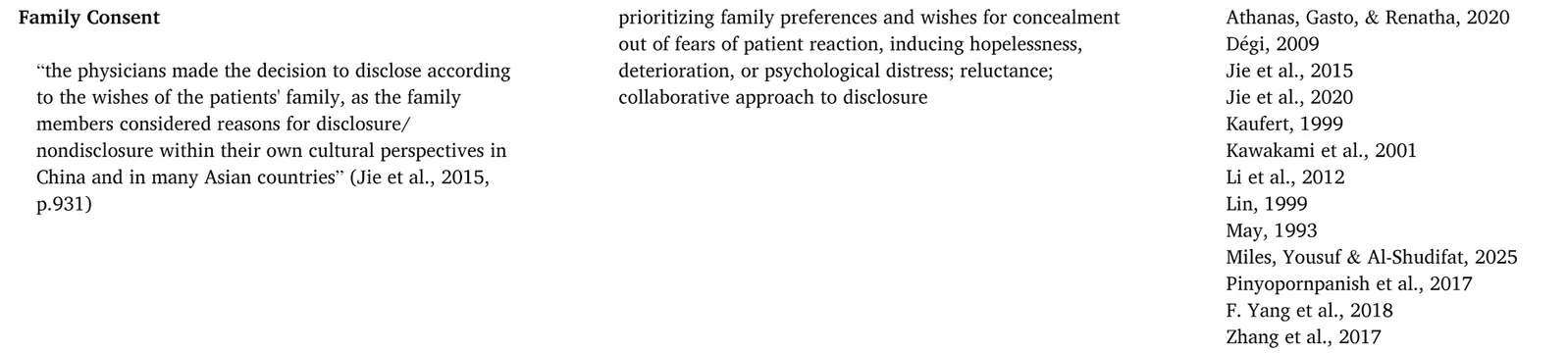
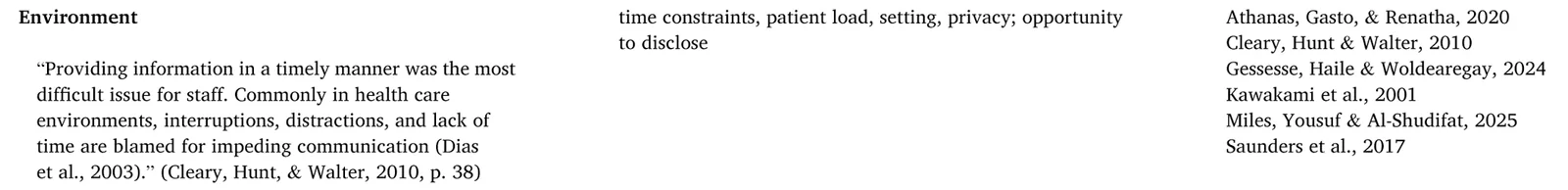
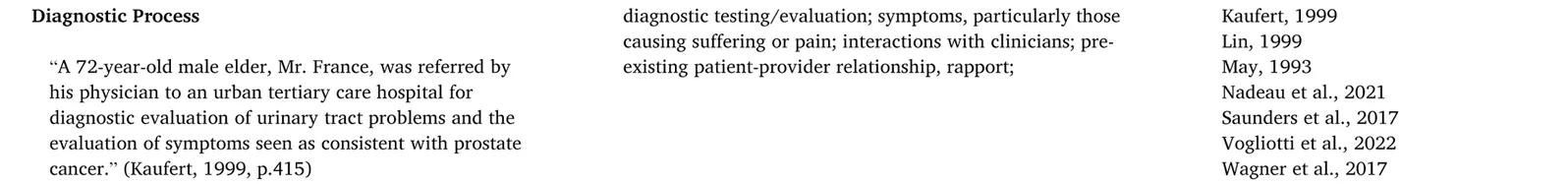
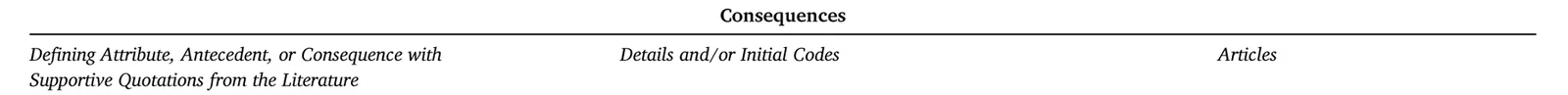
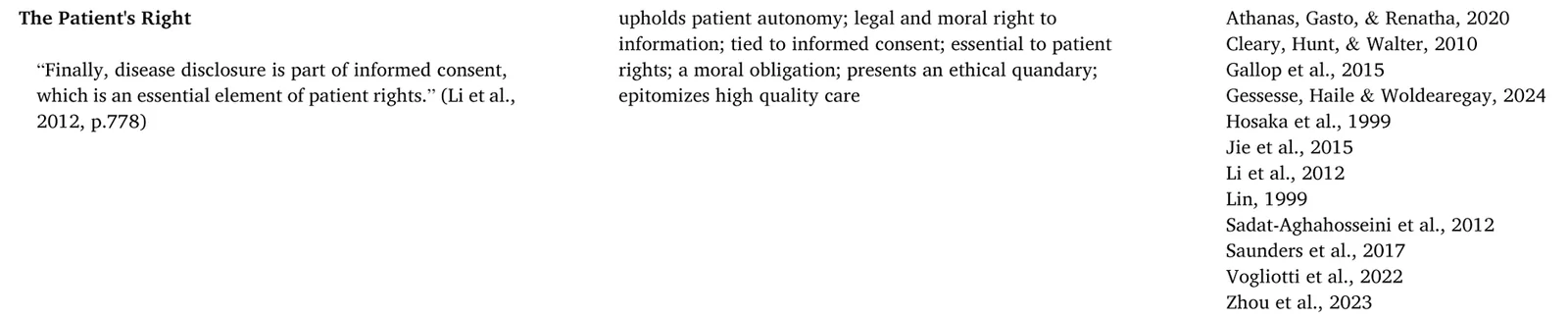
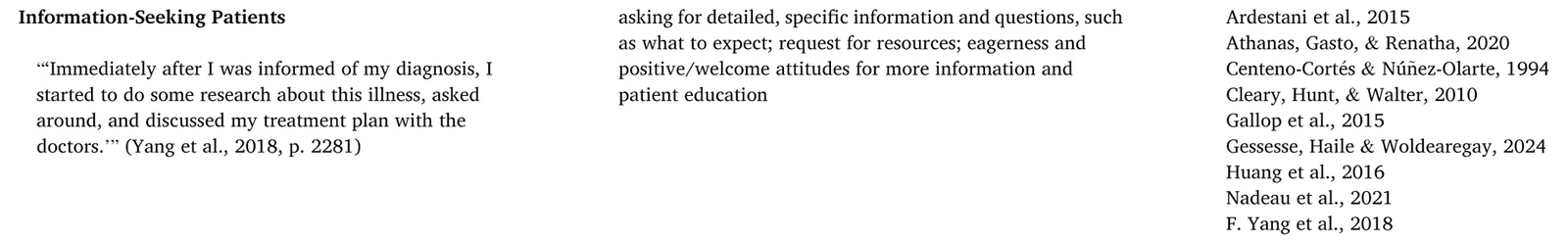
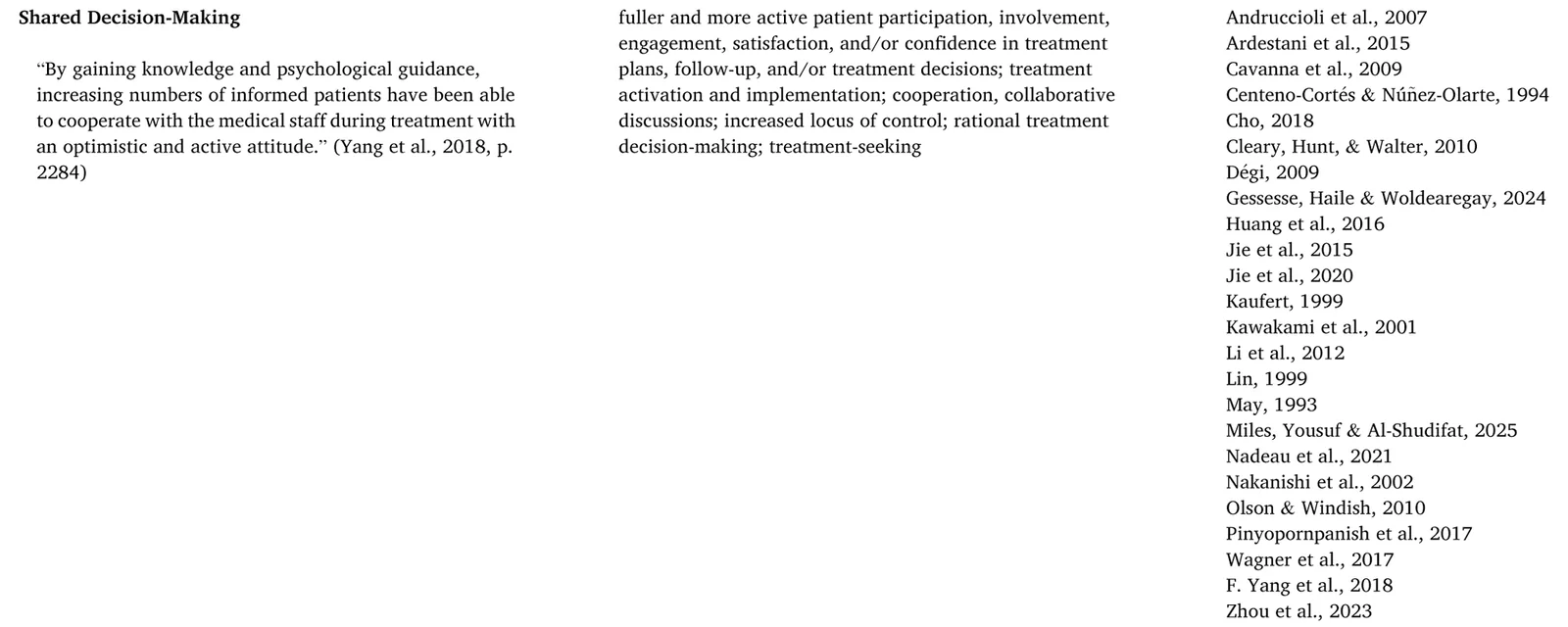
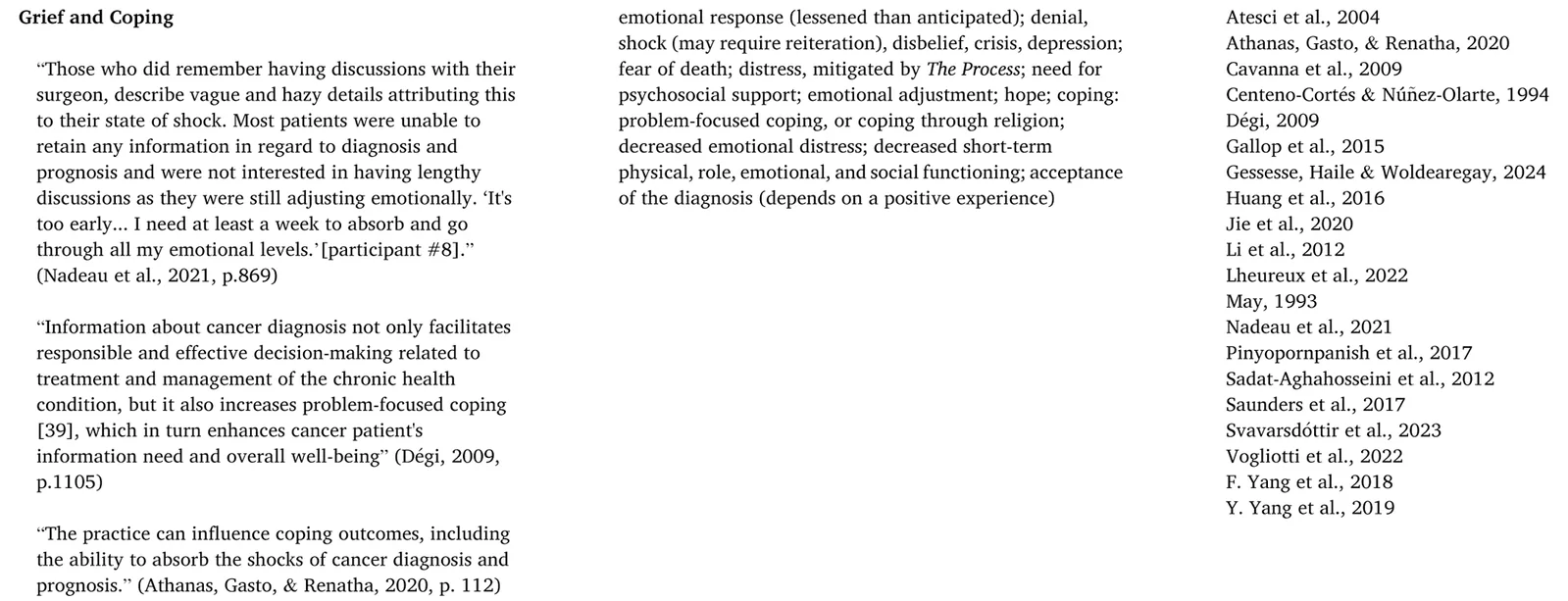
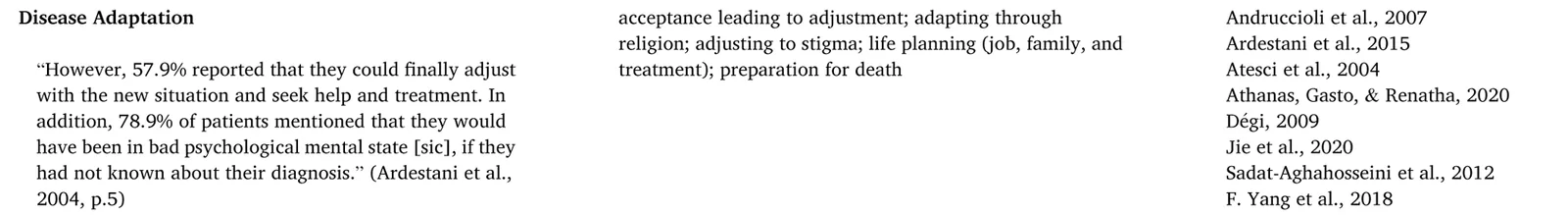
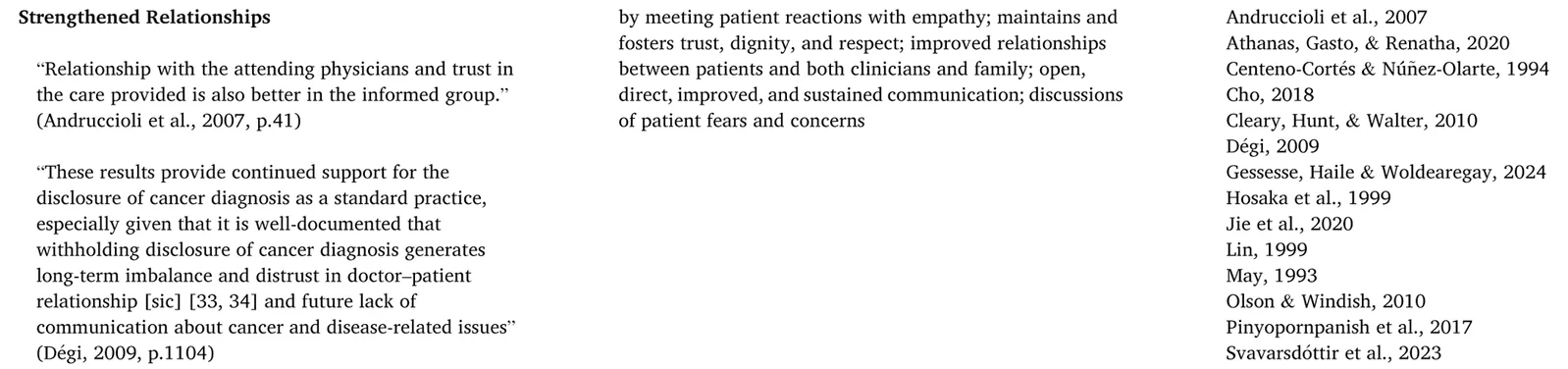
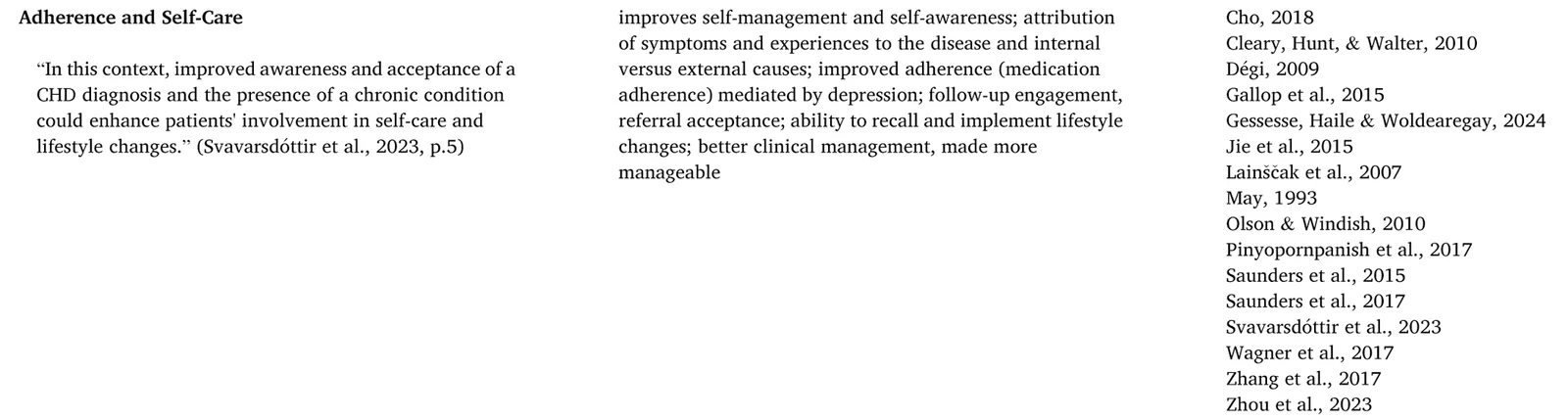
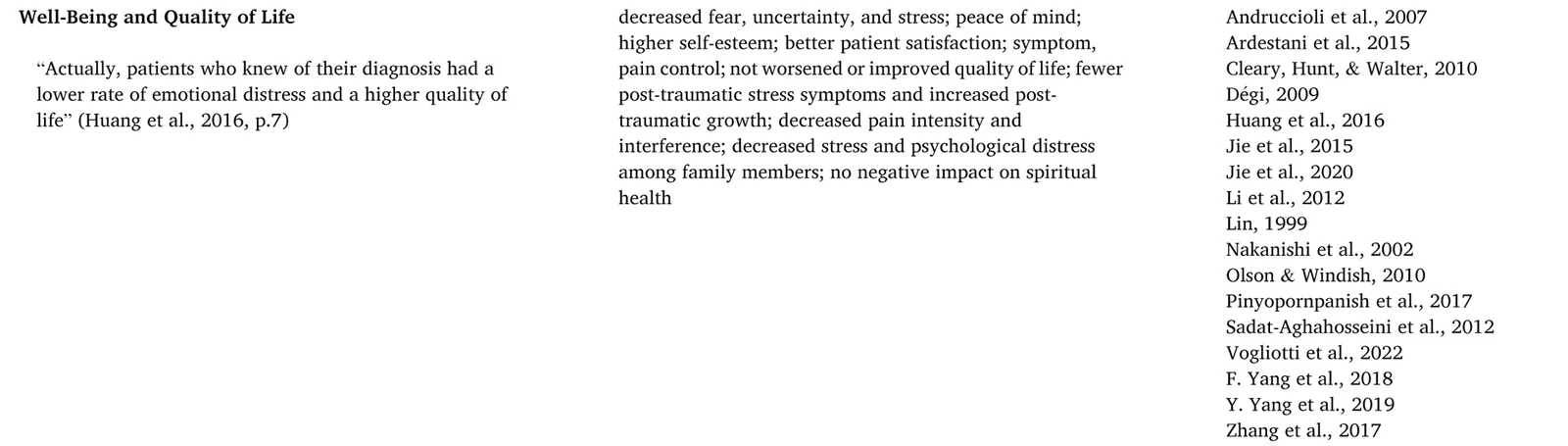

## What is already known

- Many patients are not aware of their diagnoses.
- Diagnostic disclosure lacks a clear definition in the current literature.

## What this paper adds

- Literature-driven definitions of diagnostic disclosure through two defining attributes.
- Provides relevant contextual factors (antecedents) and consequences of diagnostic disclosure.
- A novel discussion of the meaning and importance of diagnostic disclosure in the acute care setting.

## Appendix A. Supplementary data

Supplementary data to this article can be found online at https://doi.org/10.1016/j.ijnurstu.2026.105522.
