# Supplementary material for: Defining diagnostic disclosure in the acute care setting: A concept analysis
Source: Int J Nurs Stud. Author manuscript; Available in PMC 2026 Sep 3. (PMC13540972; doi:10.1016/j.ijnurstu.2026.105522)
Supplement: Supplementary file 1 [file NIHMS2168319-supplement-Supplementary_Table.docx]

| **Supplementary Table 1. Search Strategy** | | |  |  |  |  |
| --- | --- | --- | --- | --- | --- | --- |
|  | Controlled vocabulary | Limits/Restrictions | Key terms/proximity searches | Limits/Restrictions | Finalized search strategy | Results |
| PubMed | "Truth Disclosure"  "Diagnosis"  **Inpatient Setting -**   "Inpatients", "Hospitals", "Hospital Medicine" | Major Topic (Truth Disclosure and Diagnosis) | "bad news" AND diagnosis[Mesh] OR diagnosis NEAR/4 telling OR diagnostic NEAR/4 disclosure OR lack awareness NEAR/4 diagnosis OR patient awareness NEAR/4 diagnosis OR diagnosis NEAR/4 disclosure OR patient awareness NEAR/4 condition; inpatient OR hospital* | Title/Abstract;  NOT neonatal, pediatric, prenatal, maternal, or genetic;   NOT "financial disclosure" or "disclosure of interest" | (("Truth Disclosure"[Majr] AND "Diagnosis"[Majr]) OR ("bad news"[Title/Abstract] AND "Diagnosis"[Majr]) OR "diagnosis telling"[Title/Abstract:~4] OR "diagnostic disclosure"[Title/Abstract:~4] OR "patient awareness diagnosis"[Title/Abstract:~4] OR "lack awareness diagnosis"[Title/Abstract:~4] OR "patient awareness condition"[Title/Abstract:~4] OR "diagnosis disclosure"[Title/Abstract:~4]) **AND** ("Hospitals"[Mesh] OR"Hospital Medicine"[Mesh] OR "Inpatients"[Mesh] OR inpatient OR hospital*) NOT (neonatal OR pediatric OR prenatal OR genetic OR maternal OR "financial disclosure" OR "disclosure of interest") | 533 |
| CINAHL | "Diagnosis" AND "Truth Disclosure"; **Inpatient Setting -** Hospital Units+, Hospital Medicine, MH Hospitals+, Inpatients | Major Concept (Diagnosis and Truth Disclosure) | "bad news" AND diagnosis OR diagnosis NEAR/4 telling OR diagnostic NEAR/4 disclosure OR patient awareness NEAR/4 diagnosis OR diagnosis NEAR/4 disclosure OR patient awareness NEAR/4 condition  OR lack N2 awareness AND AB awareness N4 diagnosis | Abstract  NOT neonatal, pediatric, prenatal, maternal, or genetic;   NOT "financial disclosure" or "disclosure of interest" | (MM "Diagnosis" AND MM "Truth Disclosure+") OR (AB "bad news" AND MM "Diagnosis") OR AB diagnosis N4 telling OR AB diagnostic N4 disclosure OR AB patient awareness N4 diagnosis OR AB diagnosis N4 disclosure OR AB patient awareness N4 condition OR (AB lack N2 awareness AND AB awareness N4 diagnosis) **AND** (MH “Hospital Units+” OR MH “Hospital Medicine” OR MH “Hospitals+” OR MH “Inpatients”) NOT (neonatal OR pediatric OR prenatal OR maternal OR genetic OR "financial disclosure" OR "disclosure of interest") | 748 |
| Scopus | n/a | n/a | "bad news" AND "diagnosis" OR diagnosis NEAR/4 telling OR lack NEAR/2 awareness AND awareness NEAR/4 diagnosis OR lack NEAR/2 awareness AND awareness NEAR/4 condition OR diagnostic NEAR/4 disclosure OR diagnosis NEAR/4 disclosure OR "patient awareness" AND awareness NEAR/4 diagnosis OR "patient awareness" AND awareness NEAR/4 condition; inpatient OR hospital* | Title, Abstract, and/or Key Words; NOT neonatal, pediatric, prenatal, or genetic;   NOT "financial disclosure" or "disclosure of interest" | TITLE-ABS-KEY ( {bad news} AND "diagnosis" ) OR TITLE-ABS-KEY ( diagnosis W/4 telling ) OR TITLE-ABS-KEY ( diagnostic W/4 disclosure ) OR TITLE-ABS-KEY ( disclosure PRE/4 diagnosis ) OR TITLE-ABS-KEY ( {patient awareness} AND awareness W/4 diagnosis ) OR TITLE-ABS-KEY ( {patient awareness} AND awareness W/4 condition ) OR TITLE-ABS-KEY ( lack W/2 awareness AND awareness W/4 diagnosis ) AND ( hospital* OR inpatient ) AND NOT TITLE-ABS-KEY ( neonatal OR pediatric OR prenatal OR genetic OR maternal ) AND NOT TITLE-ABS-KEY ( {financial disclosure} OR {disclosure of interest} ) | 1007 |

**Supplementary Table 1, continued.**

| Embase | "diagnosis" AND "interpersonal communication"  **Inpatient Setting -**   "Hospital patient", "Hospital", "Hospital Admission" | Major Concept | "bad news" AND "diagnosis" OR diagnosis NEAR/4 telling OR lack NEAR/2 awareness AND awareness NEAR/4 diagnosis OR lack NEAR/2 awareness AND awareness NEAR/4 condition OR diagnostic NEAR/4 disclosure OR diagnosis NEAR/4 disclosure OR "patient awareness" AND awareness NEAR/4 diagnosis OR "patient awareness" AND awareness NEAR/4 condition; inpatient OR hospital* | Title, Abstract, and/or Key Words;  NOT neonatal, pediatric, prenatal, maternal, or genetic;   NOT "financial disclosure" or "disclosure of interest" | (('diagnosis'/mj AND 'interpersonal communication'/mj) OR ((diagnosis NEAR/4 telling):ti,ab,kw) OR ((diagnostic NEAR/4 disclosure):ti,ab,kw) OR ((diagnosis NEAR/4 disclosure):ti,ab,kw) OR ('patient awareness':ti,ab,kw AND ((awareness NEAR/4 condition):ti,ab,kw)) OR (((lack NEAR/2 awareness):ti,ab,kw) AND ((awareness NEAR/4 diagnosis):ti,ab,kw)) OR ('patient awareness':ti,ab,kw AND ((awareness NEAR/4 diagnosis):ti,ab,kw)) OR ('diagnosis'/mj AND 'bad news':ti,ab,kw)) **AND** ('hospital patient'/exp OR 'hospital'/exp OR 'hospital admission'/exp OR hospital* OR inpatient) NOT prenatal:ti,ab,kw NOT neonatal:ti,ab,kw NOT pediatric:ti,ab,kw NOT genetic:ti,ab,kw NOT maternal:ti,ab,kw NOT 'financial disclosure' NOT 'disclosure of interest' | 801 |
| --- | --- | --- | --- | --- | --- | --- |
